# Human immune phenotyping reveals accelerated aging in type 1 diabetes

**DOI:** 10.1172/jci.insight.170767

**Published:** 2023-09-08

**Authors:** Melanie R. Shapiro, Xiaoru Dong, Daniel J. Perry, James M. McNichols, Puchong Thirawatananond, Amanda L. Posgai, Leeana D. Peters, Keshav Motwani, Richard S. Musca, Andrew Muir, Patrick Concannon, Laura M. Jacobsen, Clayton E. Mathews, Clive H. Wasserfall, Michael J. Haller, Desmond A. Schatz, Mark A. Atkinson, Maigan A. Brusko, Rhonda Bacher, Todd M. Brusko

**Affiliations:** 1 Department of Pathology, Immunology, and Laboratory Medicine, College of Medicine, and; 2 Diabetes Institute and; 3 Department of Biostatistics, College of Public Health and Health Professions, University of Florida, Gainesville, Florida, USA.; 4 Department of Pediatrics, Emory University, Atlanta, Georgia, USA.; 5 Genetics Institute and; 6 Department of Pediatrics, College of Medicine, University of Florida, Gainesville, Florida, USA.

**Keywords:** Autoimmunity, Immunology, Autoimmune diseases, Diabetes

## Abstract

The proportions and phenotypes of immune cell subsets in peripheral blood undergo continual and dramatic remodeling throughout the human life span, which complicates efforts to identify disease-associated immune signatures in type 1 diabetes (T1D). We conducted cross-sectional flow cytometric immune profiling on peripheral blood from 826 individuals (stage 3 T1D, their first-degree relatives, those with ≥2 islet autoantibodies, and autoantibody-negative unaffected controls). We constructed an immune age predictive model in unaffected participants and observed accelerated immune aging in T1D. We used generalized additive models for location, shape, and scale to obtain age-corrected data for flow cytometry and complete blood count readouts, which can be visualized in our interactive portal (ImmScape); 46 parameters were significantly associated with age only, 25 with T1D only, and 23 with both age and T1D. Phenotypes associated with accelerated immunological aging in T1D included increased CXCR3^+^ and programmed cell death 1–positive (PD-1^+^) frequencies in naive and memory T cell subsets, despite reduced PD-1 expression levels on memory T cells. Phenotypes associated with T1D after age correction were predictive of T1D status. Our findings demonstrate advanced immune aging in T1D and highlight disease-associated phenotypes for biomarker monitoring and therapeutic interventions.

## Graphical abstract

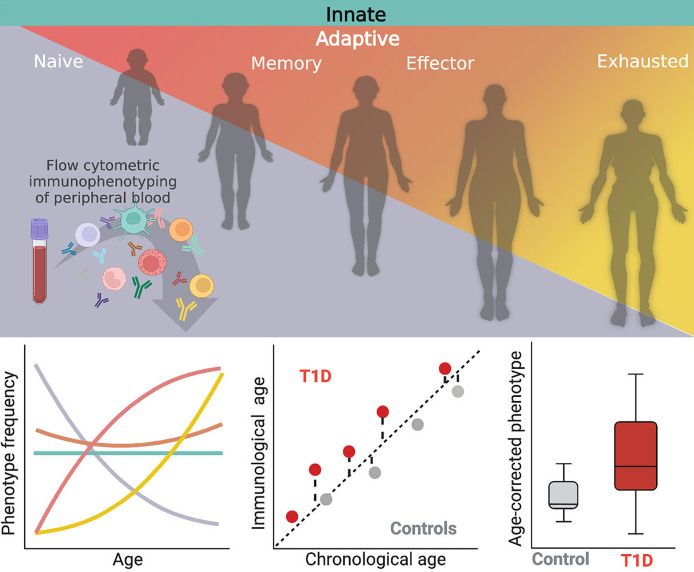

## Introduction

With improved diabetes classification tools, it is now appreciated that the onset of type 1 diabetes (T1D) may occur throughout the human life span (1, 2), though diagnosis peaks between ages 5 to 7 years and near puberty (3). Individuals with high genetic risk for T1D, which is largely driven by the human leukocyte antigen (HLA) region, are more likely to receive diagnoses during these peak time periods (4, 5) and exhibit islet cell–reactive autoantibodies (AAbs) indicative of disease progression before age 2 (6, 7). Additionally, the cellular composition of insulitis has been demonstrated to depend upon the age at onset of T1D (5, 8), with a shift from B cell–dominated insulitis seen under age 7 to primarily T cells and macrophages over age 13 (9, 10), suggesting that the immune populations involved in disease pathogenesis vary by age at diagnosis.

The quest for cellular biomarkers of T1D pathogenesis is limited to recirculating immune cells that do not perfectly reflect the populations in priming lymph nodes and autoimmune lesions (11) and is further confounded by age- and environment-driven variations in peripheral blood immune cell subset composition (12). To address these confounding technical and biological factors masking disease-related changes in the immune system, the Human Immunophenotyping Consortium (HIPC) developed a recommended set of flow cytometry panels aiding aggregation and comparison of data across studies (13). The HIPC panels were designed to quantify memory T cell, regulatory T cell (Treg), effector T cell, B cell, dendritic cell (DC), monocyte, and natural killer (NK) cell subset proportions and phenotypes (13). These standardized panels have been successfully used to identify immune modulation due to vaccination (14, 15), infection (16, 17), autoimmunity (18, 19), and cancer (20, 21), though to our knowledge, full HIPC phenotyping of T1D has not been previously performed.

The impact of aging on immune phenotypes is well established (22, 23); environmental exposures, particularly infection by CMV, are known to drive expansion of a large circulating pool of antigen-specific memory T cells (24, 25). However, previous investigations have primarily been limited to cohorts of adults. Here, we studied a more expansive age range (2–83 years) to capture the immune dynamics of childhood and adolescence, wherein the majority of T1D diagnoses occur. In order to study disease-mediated perturbations of the immune system, our cross-sectional cohort (*n* = 826) was designed to include unaffected controls (CTR, *n* = 252), unaffected first-degree relatives of individuals with T1D (REL, *n* = 310), rare at-risk participants who have 2 or more islet AAbs (RSK, *n* = 24), and those diagnosed with T1D (*n* = 240).

In this study, we categorized major patterns of immune subset trajectories in unaffected individuals over the pediatric and adult age range, allowing for detection of modulated trajectories in T1D. We further modeled the immunophenotyping data to estimate immunological age (26) as compared with chronological age in individuals with T1D versus those without diabetes. Age-corrected individual phenotypes contributing to differences in immunological age were compared across all participants binned by progressive T1D risk or status (27) to understand whether each was likely to contribute to immune activation and disease pathogenesis as opposed to a consequence after onset (e.g., dysglycemia-induced inflammation; ref. 28). Last, we assessed associations between T1D genetic risk (29–31) and immunophenotypes and found limited genetic associations with accelerated immune aging in T1D. Notably, all immunophenotyping data generated herein are available for visualization and analysis via an interactive R/Shiny application (ImmScape; https://ufdiabetes.shinyapps.io/ImmScape/).

## Results

### Impact of age on immune population dynamics.

**Figure 1:**
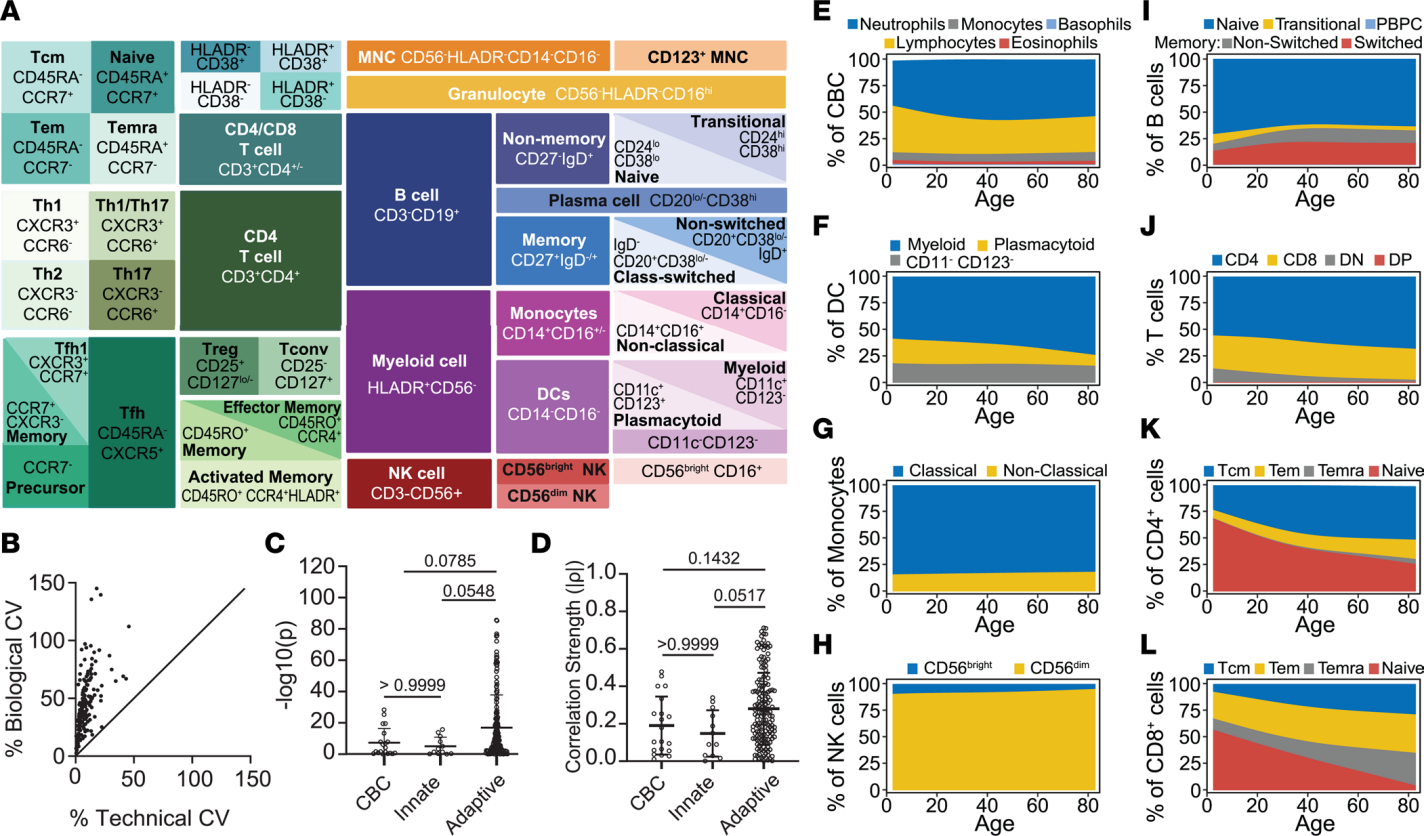
Immune population dynamics and QC of outcome measures. (**A**) Schematic representation of hierarchical gating strategy used to identify 172 immune cell subsets evaluated from human peripheral blood. An additional 20 parameters were derived from CBC. (**B**) Low technical variation observed from peripheral blood samples (*n* = 12) stained in duplicate for assessment by flow cytometry. (**C**) –log10(p) (**D**) and correlation strength (absolute value of ρ) from Spearman’s correlation between phenotypes (each phenotype is a data point) and age showing strongest associations in the adaptive compartment as compared with innate or CBC. Kruskal-Wallis test with Dunn’s multiple-comparison test results denoted above bars. (**E**–**L**) Phenotype proportions estimated using a smoothing spline model as a function of age in AAb- individuals. (See Supplemental Figures 1–6 and Supplemental Tables 1 and 7.) QC, quality control; CBC, complete blood count; Tcm, T central memory; Tem, T effector memory; Temra, T effector memory CD45RA^+^; Tfh, T follicular helper; Tconv, T conventional; MNC, mononuclear cells; PBPC, plasmablasts/plasma cells; DN, double negative; DP, double positive.

We used 6 flow cytometry panels adapted from HIPC recommendations (13), as previously published (18, 32), to generate detailed immunophenotyping data encompassing proportions of innate and adaptive immune cells (Figure 1A, detailed gating in Supplemental Figures 1–6; supplemental material available online with this article; https://doi.org/10.1172/jci.insight.170767DS1). Assay reproducibility was demonstrated in an initial cohort of 12 individuals wherein the biological coefficient of variance (CV) largely outweighed variance between technical duplicates (45.23% ± 25.66% vs. 8.87% ± 7.56%, Figure 1B), in agreement with previously established guidelines for replicability in flow cytometric studies (33). These studies were then extended to characterize flow cytometric immunophenotypes with accompanying CBC measurements, for a total of 192 total outcome measures, on a large cross-sectional cohort of *n* = 826 persons (Table 1). The majority of CBC values were within normal range, with the following exceptions: low mean corpuscular hemoglobin concentration, low neutrophil percentage, and high lymphocyte percentage were observed across CTR, REL, and T1D groups, presumably related to transport and storage time (Supplemental Table 1). The T1D group displayed increased proportions of individuals with hematocrit percentage and platelet count above the normal range, potentially reflecting dehydration and hyperglycemia, respectively (34) (Supplemental Table 1). Covariates including age, sex, BMI percentile, and race differed between groups (27) (Table 1). The AAb- and T1D cohorts both had bimodal age distributions with different proportions in each component, as is common in pediatric T1D cohorts with unaffected individuals comprised largely of siblings and parents. Upon noting this age discordance, we quantified how each immune phenotype changed with age. We performed Spearman’s correlation analyses between age and all peripheral blood phenotypes obtained by CBC and flow cytometry in AAb-negative (AAb-) unaffected individuals (CTR and REL), demonstrating that the majority of age-related associations appeared in the adaptive immune compartment (Figure 1, C and D). Visualization of the dynamics of major subsets defined by our flow cytometry panels revealed relatively consistent proportions of innate cells across age, including monocytes, DCs, and NK cells, in contrast to distinct age-related contraction of naive and expansion of memory subsets within the adaptive B cell and T cell compartments (Figure 1, E–L).

### Age-associated immune trajectories in T1D.

**Figure 2:**
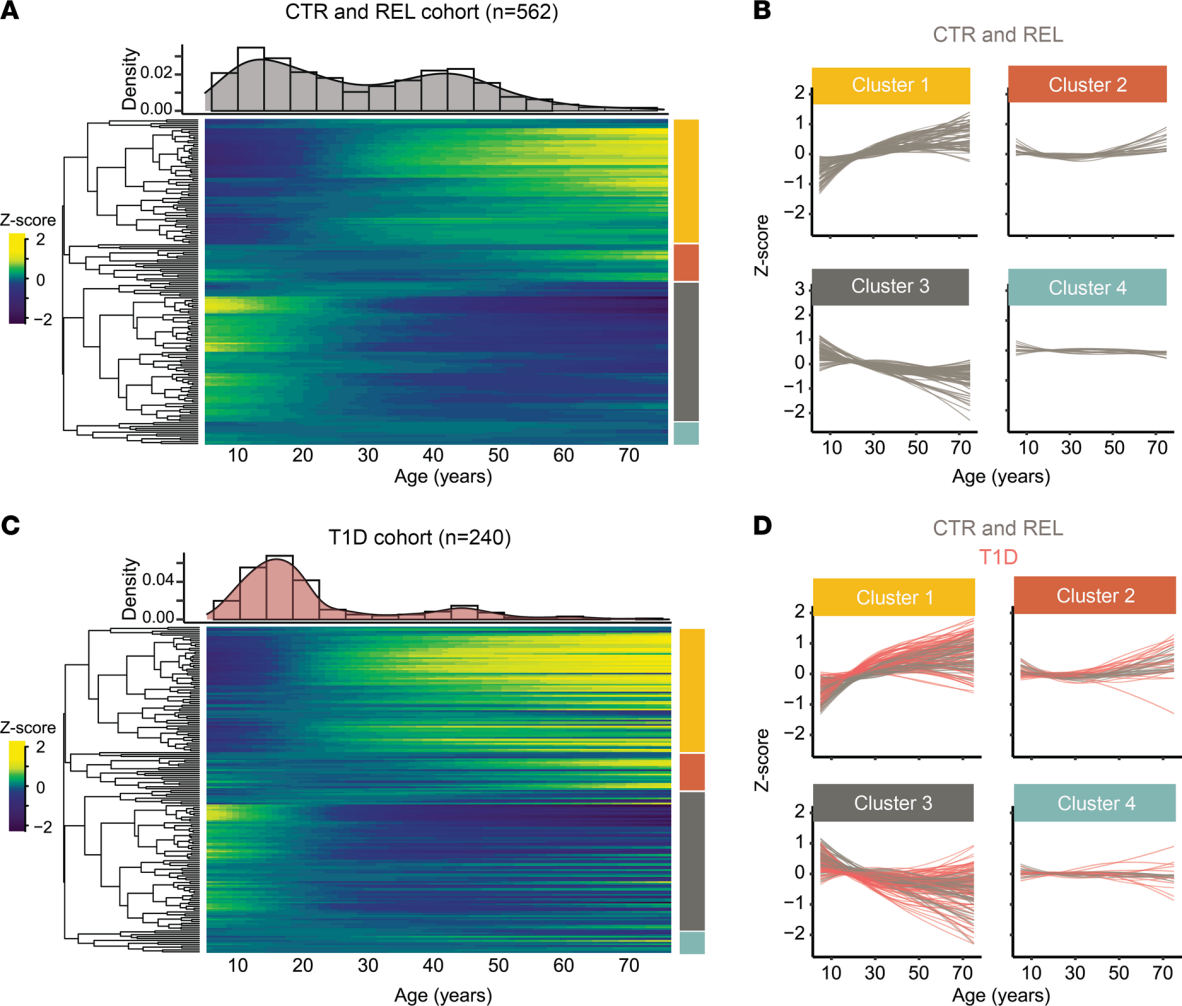
Immunophenotype trajectories in T1D. (**A**) Heatmap of smoothed phenotype trajectories as a function of age in AAb- individuals with analysis restricted between the ages of 5 and 75 years to avoid predicting from sparse data. The age distribution of the cohort within this age range is shown (top histogram). Immune cell phenotypes were clustered into 4 distinct groups (axis colors, right) using hierarchical clustering (dendrogram, left). (**B**) Line plots of each smoothed phenotype as a function of age demonstrate distinct dynamic behavior within the 4 clusters. (**C**) Heatmap of smoothed phenotype trajectories as a function of age in T1D individuals with the rows arranged as in **A**. (**D**) Line plots of each smoothed phenotype as in **B** with the T1D smoothed phenotypes overlaid in red. (See Supplemental Figures 7 and 8.) Shifts in cluster trajectories for T1D versus AAb- were compared using a 2-tailed *t* test (cluster 1, *P* < 0.001; cluster 3, *P* = 0.034).

To assay the impact of age on the composition of the peripheral immune system, we characterized major trajectory patterns of the 172 flow cytometry outcome measures in unaffected islet AAb- CTR and REL 5–75 years of age. Smoothing splines were fit to model each phenotype’s trajectory over age. Hierarchical clustering analysis of normalized, centered, and scaled phenotype trajectories (Figure 2A) revealed 4 distinct patterns: 1) increasing linear (*n* = 66 phenotypes), 2) upward parabolic (*n* = 20), 3) decreasing linear (*n* = 74), and 4) stable (*n* = 12) relationships with age (Figure 2B). Using these defined cluster assignments (Supplemental Figure 7), we also fit smooth trajectories on T1D samples and overlaid these on AAb- CTR and REL (Figure 2, C and D). When organized using the clustering structure from the unaffected individuals, the T1D immune trajectories appeared to have similar overall trends (Figure 2C). Indeed, trajectory-specific difference revealed that the vast majority (86%) of phenotype trajectories initially trended in the same direction (increasing or decreasing), and those with differing initial trends crossed at least twice, indicating sampling variation in the trajectory rather than disease-driven divergence. However, direct comparison revealed an average upward shift in cluster 1 (*P* < 0.001) and a downward shift in cluster 3 (*P* = 0.034, Figure 2D) for T1D versus AAb- trajectories. Over time, trajectories tended to shift farther apart (Supplemental Figure 8), together suggesting that individuals with T1D exhibit distinct age-dependent alterations in immune trajectories.

### Accelerated immune aging in T1D.

**Figure 3:**
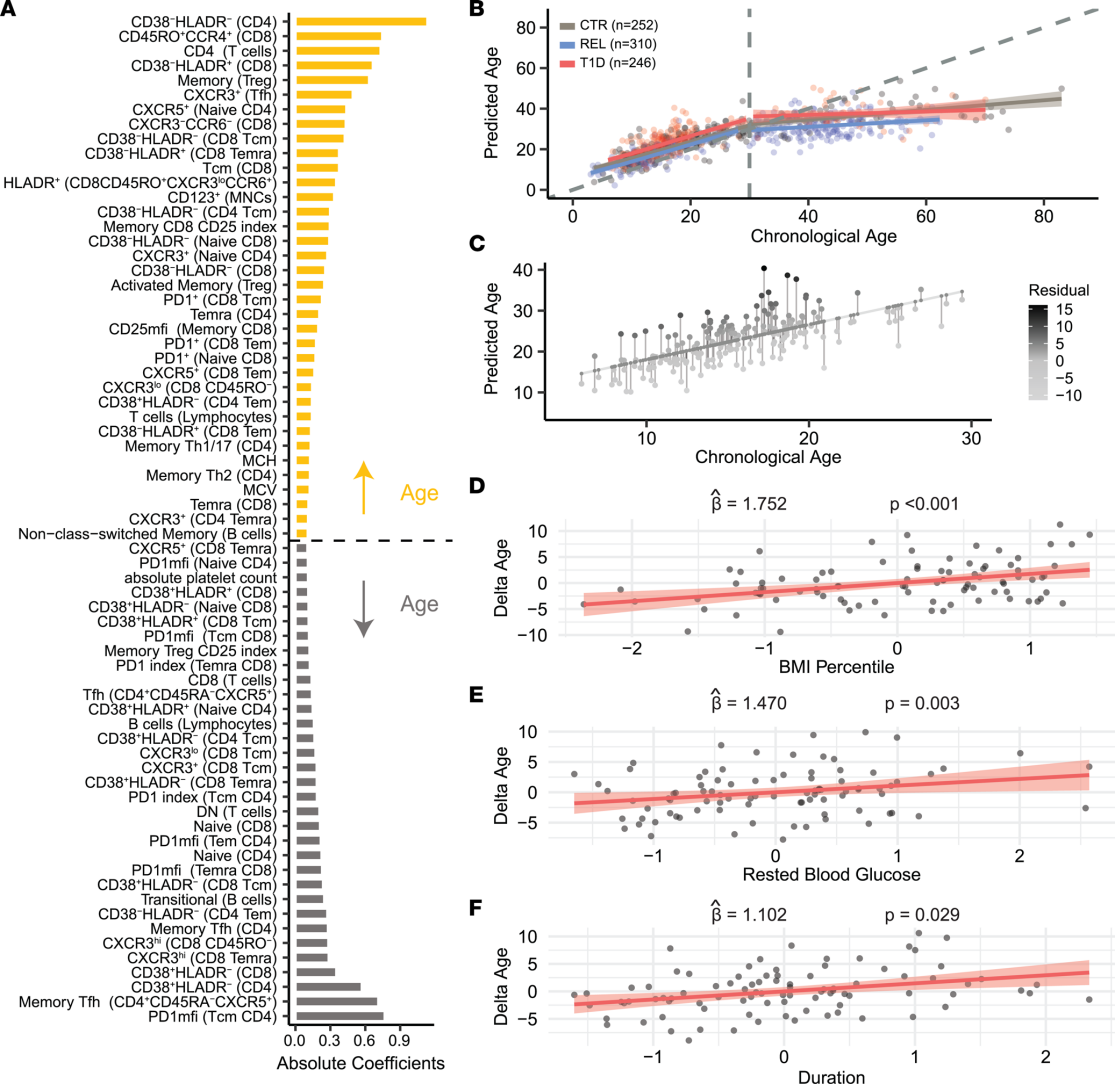
Immunophenotype age modeling reveals accelerated aging in T1D. (**A**) Averaged coefficients from the random lasso model for all phenotypes above an empirically estimated threshold, showing those increasing with age (yellow) and decreasing with age (gray). (**B**) The random lasso model was used to estimate immunological predicted age in CTR (gray), T1D (red), and REL (blue). The correspondence of predicted age with chronological age is shown using a piece-wise regression model with a break at chronological age 30. (**C**) Residual immunological age is calculated from a linear regression of predicted age and chronological age (<30 years, *n* = 193). Partial regression plots between residual age and (**D**) BMI percentile, (**E**) T1D duration, and (**F**) rested blood glucose are shown for the multivariable regression model, along with the standardized coefficient and *P* value (<30 years of age, *n* = 90). (See Supplemental Table 3, Figure 5, and Supplemental Figures 10 and 11.)

To quantify the extent to which T1D immune profiles deviate from unaffected individuals across chronologic age, we created a model to estimate an “immunological age” parameter from our CBC and flow cytometry data. Due to hierarchical dependence and correlation among flow cytometry readouts (Supplemental Figure 9), we trained a random lasso (35) model on islet AAb- CTR to identify phenotypes predictive of age in individuals without autoimmunity. Our approach retained 69 immune features (Figure 3A) with a test set prediction performance of *R*^2^ = 0.70. Model consistency was observed by training on a combined AAb- cohort in which 45 variables were commonly retained and *R*^2^ = 0.70. Providing support for this immune age model, we saw expected shifts from naive to memory populations in both T and B cells over the life span (36–38). CD4^+^ T cells increased concomitant with a decline in CD8^+^ T cell frequency, consistent with the CD4/8 ratio increasing with age (39).

We then applied our model to the full AAb- CTR, AAb- REL, and T1D cohorts to examine differences in predicted age and chronological age. We observed the highest predictive performance of the immune age model among younger individuals regardless of risk cohort, which we validated by training 2 separate random lasso models for AAb- CTR younger than the age of 30 (*R*^2^ = 0.66) and those older (*R*^2^ = 0.56). Given that the model plateaued after age 30 (Figure 3B), we focused the rest of the analysis for Figure 3 on participants under 30 years of age (Supplemental Table 2), where we observed the T1D group displayed accelerated immune aging relative to both AAb- CTR and REL, with an average increase of 3.36 years (*P* < 0.001, Figure 3B). Immunological age was increased by 1.71 years on average (*P* = 0.016) in the recent-onset T1D subset of our cohort under 30 years of age (Supplemental Figure 10A), consistent with observations in the overall T1D group. The observed accelerated immune aging in T1D did not appear to be related to history of CMV infection, which, perhaps surprisingly (24, 40), was not a significant driver of accelerated immune aging in our young cohort (average increase of 1.62 years associated with CMV infection history, *P* = 0.086, Supplemental Figure 10, B and C). We also explored 9 disease-relevant features for potential associations to the residual predicted age in T1D (Figure 3C). The average increase of 3.36 years was not associated with HbA1c, polygenic T1D risk (GRS1; ref. 5), or demographic variables such as sex, race, or ethnicity (Supplemental Table 3). Disease duration and clinical covariates BMI percentile and rested blood glucose were significantly associated with accelerated immune aging in T1D (*P* = 0.003, *P* < 0.001, and *P* = 0.029) (Figure 3, D–F) but not in AAb- CTR or REL (Supplemental Figure 11). The standardized regression coefficients in a multivariable regression model of predicted age indicated that BMI percentile had the largest contribution (β = 1.75), followed by disease duration (β = 1.47) and rested blood glucose level (β = 1.10). The increased residual age variations observed, and their association with clinical features, reflect the additional immunological burdens of T1D.

### Age correction of immunophenotyping data in a T1D prediction model.

**Figure 4:**
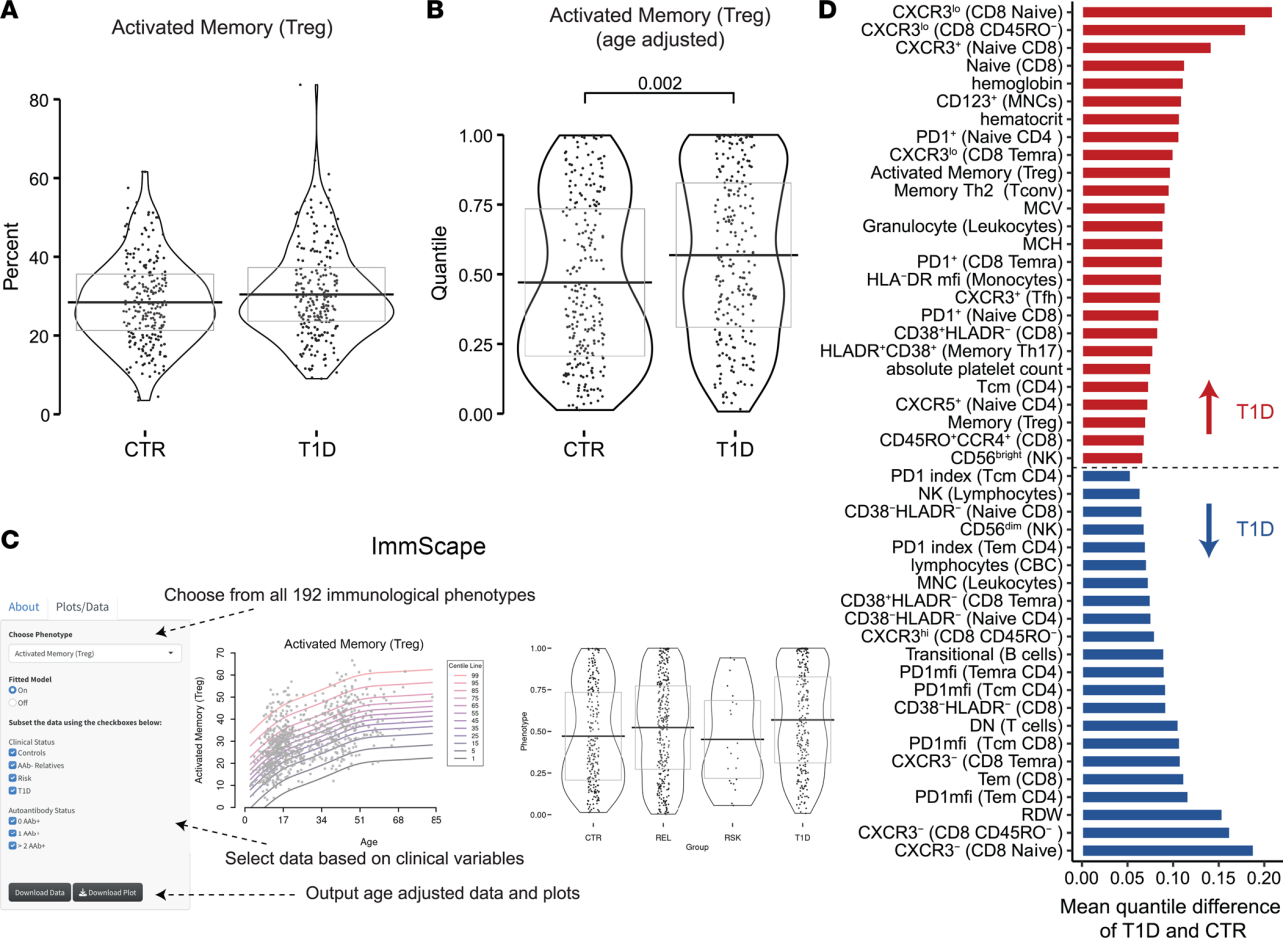
Age-corrected phenotypes reveal T1D-specific differences. As an example of the utility of our model, (**A**) in uncorrected data, there is no significant difference detected between T1D (*n* = 232) and CTR (*n* = 240). (**B**) Using the GAMLSS-corrected data, there is a significant difference between T1D and CTR. (**C**) All age-corrected data are available for download and analysis via the ImmScape R/Shiny application. (**D**) Age-corrected quantile values for T1D versus CTR were compared using nonparametric Kruskal-Wallis test and post hoc Dunn’s test with Benjamini-Hochberg multiplicity adjustment. Phenotypes increased (red) and decreased (blue) in T1D (regardless of age) are shown. (See Supplemental Table 4, Figure 5, and Supplemental Figures 9–11.) MCV, mean corpuscular volume; MCH, mean corpuscular hemoglobin; RDW, red cell distribution width; PD1, programmed cell death 1.

Given the diverse and heterogeneous phenotypic distributions across the 192 flow and CBC measures, along with their nonlinear association with age, we chose to use a semiparametric modeling framework to investigate the specific flow cytometric readouts contributing to the accelerated immune aging observed in T1D. We obtained age-corrected phenotype data for all further analyses by using generalized additive models for location, shape, and scale (GAMLSS) (41, 42). Using this approach, we modeled each phenotype as a smooth function of age using the combined AAb- CTR and REL cohorts, obtained fitted distribution parameters for all ages, and then obtained the centiles of their phenotypes from the estimated cumulative distribution function. The advantage of this approach is that in addition to age-adjusted comparison testing, it provides the framework for an immunophenotype centile reference range. We built an R/Shiny user interface (ImmScape; https://ufdiabetes.shinyapps.io/ImmScape/) for interactive exploration and visualization of study data as illustrated in Figure 4, A–C. After applying GAMLSS age correction, none of the phenotypes associated with BMI percentile or T1D duration using FDR correction for multiple testing. With these age-corrected data, we were able to identify 48 total features that were significantly different in T1D as compared with CTR participants following multiple-testing correction (26 increased and 22 decreased) (Figure 4D and Supplemental Table 4), including features from all flow cytometry panels and CBC data (Supplemental Figure 9 and Supplemental Table 1). To quantify the usefulness of the age adjustment in identifying disease-relevant phenotypes, we used logistic regression to predict disease status (T1D versus AAb- CTR) from the 48 phenotypes and obtained an area under the receiver operating characteristic curve (AUROC) of 82.3%. This is an improvement over similarly constructed prediction models on all age-corrected phenotypes (AUROC = 79.6%) and on all uncorrected phenotypes (AUROC = 76.8%).

Our immune age model also shows consistency with the previously developed IMM-AGE score in Alpert et al. (26), which fits a trajectory through a low dimensional representation of the data and estimates a pseudotime or relative order of individuals as an indicator of immune aging. Applying the IMM-AGE procedure to all 192 phenotypes, we observed a similar pattern of increased correlation between biological age and estimated pseudotime before age 30, followed by no correlation at later ages. We also found T1D individuals were shifted significantly in pseudotime relative to CTR (*P* = 0.022; Supplemental Figure 12A). When restricting our analysis to only the phenotypes identified by our random lasso model, the consistency between the analyses grew stronger, with a Spearman correlation of 0.92 between the IMM-AGE score and our predicted immune aging (Supplemental Figure 12B). However, it is important to acknowledge that the IMM-AGE score provides only a relative age value and does not correspond to a predicted age. In contrast, our model directly estimates an immune age while identifying relevant phenotypes and their impact on immune aging.

### Immune features influenced by age and T1D status.

**Figure 5:**
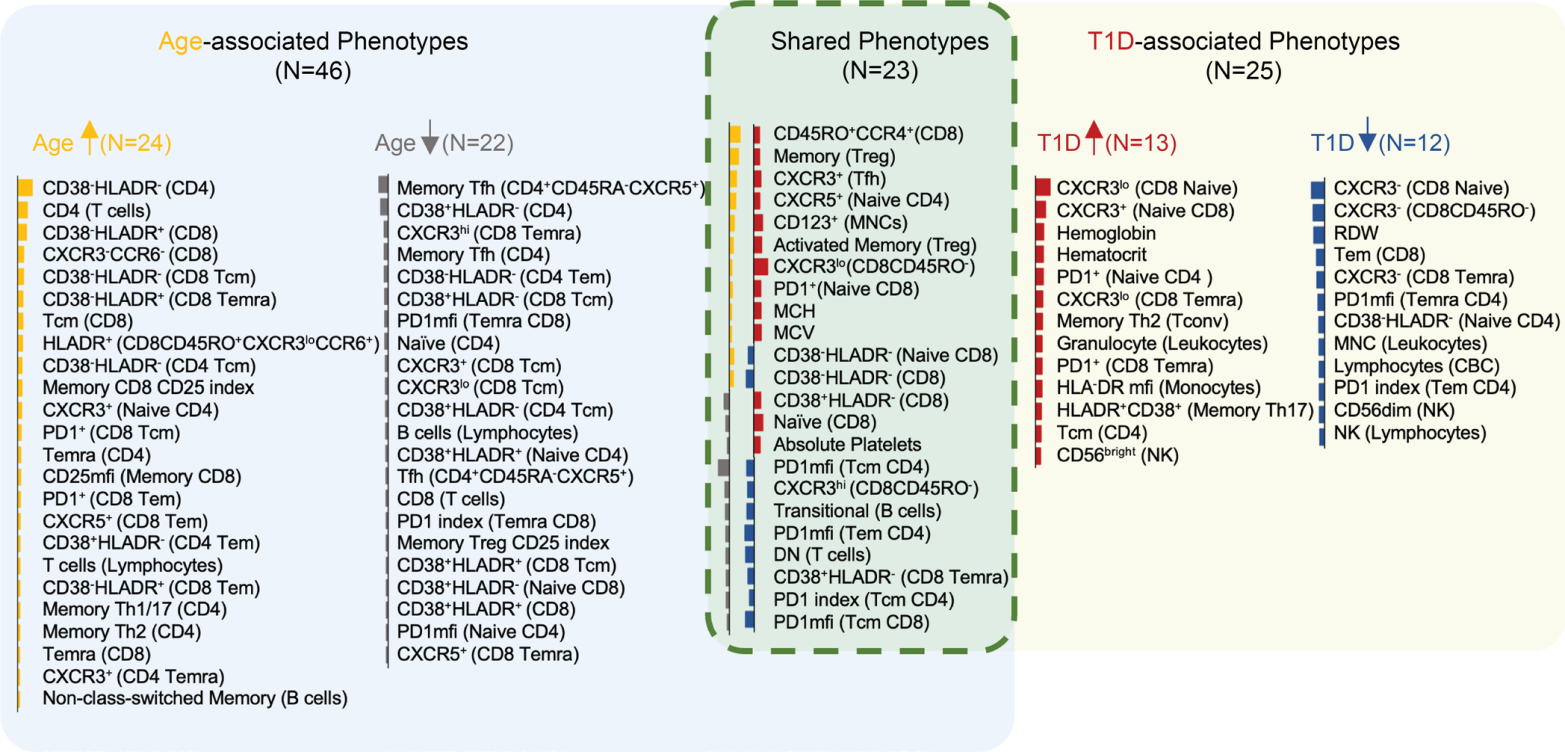
Age- and T1D-associated phenotypes. Rectangular Venn diagram summarizes phenotypes with unique association to age (left, blue shading) or T1D (right, yellow shading), with common phenotypes displayed in the overlapping area (center, green shading). The total number of phenotypes that are “unique” or “common” to age or T1D are indicated in parentheses. A color bar illustrating the magnitude and direction of effect for age or T1D is to the left of each phenotype (bar length represents the effect size; bar color indicates the phenotype is upregulated [yellow or red] or downregulated [gray or blue] in age or T1D, respectively). (See Figure 3A and Figure 4D.)

Together, 46 features were significantly modulated with age alone versus 25 with T1D status, while 23 features had shared contributions from both age and T1D (Figure 5). Several observations within our age-corrected data validate previously reported findings in T1D. For example, the age-corrected proportion of naive CD8^+^ T cells increased, while the CD8^+^ Tem cell population exhibited decreased age-corrected frequency in T1D (43) (Supplemental Figure 13, A and B). We observed reduced frequencies of CD8^+^ T cells lacking both activation markers CD38 and HLA-DR in T1D with concomitant increase in CD8^+^CD38^+^HLA-DR^–^ cells (44) (Supplemental Figure 13, C and D). CD56^dim^ NK cells decreased with expected concomitant increase in CD56^bright^ NK cells in T1D participants, in agreement with 2 publications (44, 45), but in conflict with another (46), as well as a trend (*P* = 0.095) in ≥2AAb+ RSK participants (Supplemental Figure 13E). Furthermore, T1D participants showed reduced frequency of transitional B cells as compared with AAb- CTR and REL (Supplemental Figure 13F) (47). As summarized in Figure 5, we found an association of increasing non-class-switched memory B cells with age, while transitional B cells declined with both aging and T1D (48, 49). Finally, CBC parameters showed both age- and T1D-dependent shifts: MCV and MCH increased with age and T1D, while hemoglobin and hematocrit increased in T1D, findings supported by existing literature (50). With the above supporting our approach, we identified themes pertinent to immune dysregulation in T1D: altered expression of the Th1-associated chemokine receptor CXCR3 and coinhibitory receptor PD-1 on multiple T cell subsets, as well as increased monocyte expression of HLA-DR (Figure 5), which we explored further as described below.

### Increased CXCR3 expression on T cell subsets of T1D participants.

**Figure 6:**
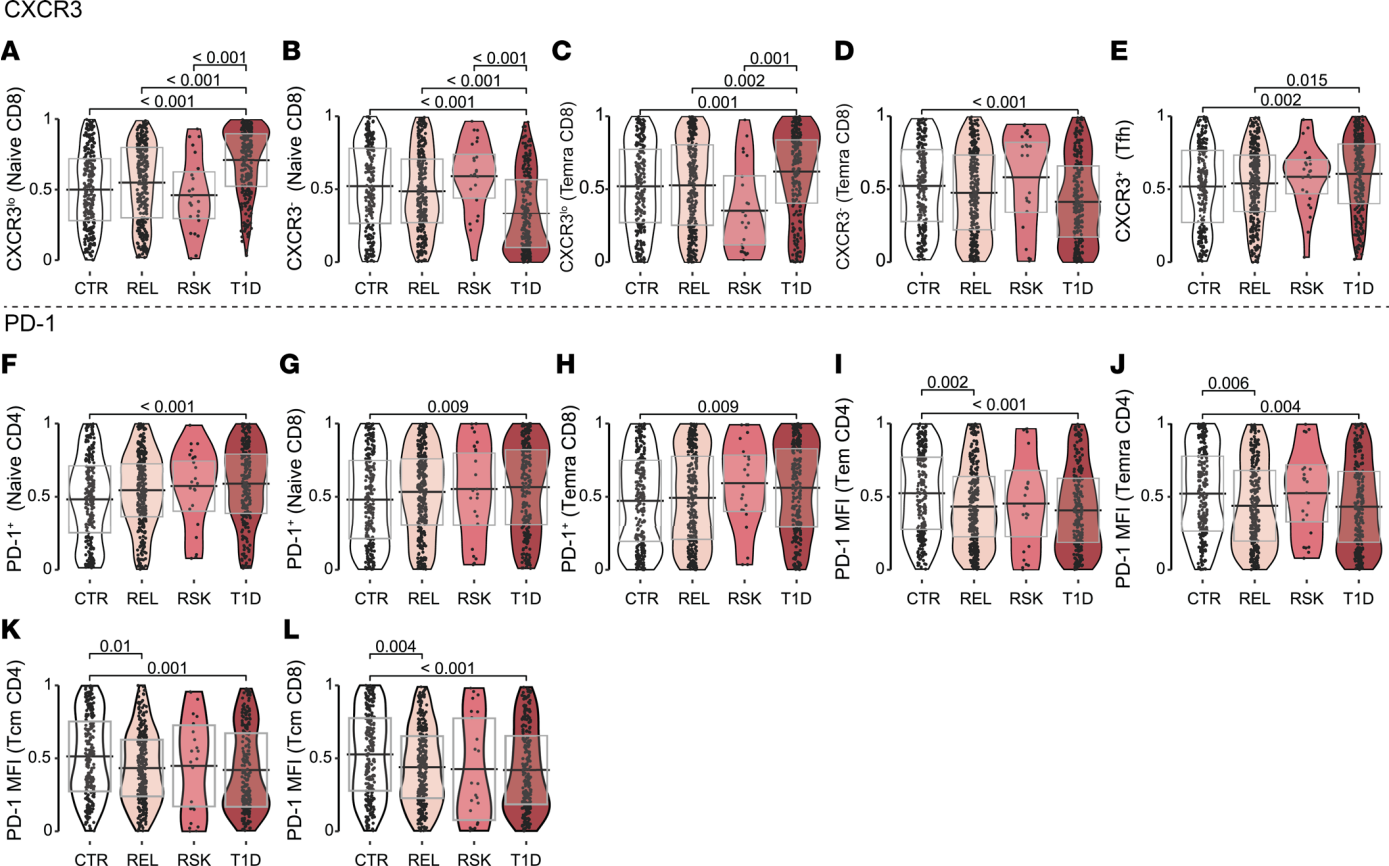
Increase in CXCR3-expressing T cell subsets and increased frequency, albeit lower intensity, of PD-1 expression in T1D. Age-corrected quantile values for (**A**–**E**) CXCR3^lo^, CXCR3^–^, or CXCR3^+^ frequency; (**F**–**H**) PD-1^+^ frequency; and (**I**–**L**) PD-1 MFI on T cell phenotypes (CTR *n* = 240, REL *n* = 293, RSK *n* = 23, T1D *n* = 232) for **A**–**D**; (CTR *n* = 247, REL *n* = 299, RSK *n* =24, T1D *n* = 235) for **E**; (CTR *n* = 247, REL *n* = 298, RSK *n* = 23, T1D *n* = 237) for **F**–**L**. Significant *P* values shown on graph (Kruskal-Wallis test with post hoc Dunn’s test and Benjamini-Hochberg multiplicity adjustment). (See Supplemental Figure 14.)

Of all parameters measured, the greatest mean difference between T1D and CTR (without a significant age association) was substantially increased frequency of CXCR3 expression among naive CD8^+^ T cells (Figure 5), due to increased CXCR3^lo^ and decreased CXCR3^–^ subset percentages (Figure 6, A and B, and Supplemental Figure 14, A and B). CD8^+^ Temra exhibited the same patterns of increased CXCR3^lo^ and decreased CXCR3^–^ frequencies in T1D (Figure 6, C and D, and Supplemental Figure 14, C and D). Individuals with T1D also demonstrated elevated frequencies of CXCR3^+^ Tfh (Figure 6E and Supplemental Figure 14E) as previously reported (44). Together, these data show a shift toward increased CXCR3-expressing populations across T cell subsets in T1D.

### Altered PD-1 expression on T cells from T1D participants.

We observed altered expression of the coinhibitory receptor, PD-1, on T cell subsets (Figure 5). Despite significantly increased frequency of PD-1^+^ cells within naive CD4^+^, naive CD8^+^, and CD8^+^ Temra subsets from T1D participants (Figure 6, F–H, and Supplemental Figure 14, F–H), PD-1 expression intensity (MFI) was decreased in T1D participants on the majority of subsets analyzed: CD4^+^ Tem, CD4^+^ Temra, CD4^+^ Tcm, and CD8^+^ Tcm (Figure 6, I–L, and Supplemental Figure 14, I–L). Importantly, due to age-associated changes in PD-1 expression across all clinical groups (Figure 3A), a number of these T1D-associated differences were most apparent following age correction (Figure 6, G and J–L, and Supplemental Figure 14, G and J–L). Interestingly, PD-1 MFI was also significantly decreased on memory CD4^+^ and CD8^+^ T cell subsets of AAb- REL as compared with CTR (Figure 6, I–L, and Supplemental Figure 14, I–L), suggesting a potential genetic predisposition to altered expression of PD-1.

While some studies in small, racially homogenous cohorts have associated single nucleotide polymorphisms (SNPs) in the *PDCD1* locus with T1D (51–54), large genome-wide association studies (GWAS) in European cohorts have failed to replicate these findings (31, 55). We surmised that a *PDCD1* SNP that has not been identified in GWAS efforts may be enriched in T1D, AAb+, and AAb- REL participants from our trans-ancestral cohort (Table 1). To test this hypothesis, we downloaded *PDCD1* expression quantitative trait loci for whole blood from the Genotype-Tissue Expression project (GTEx) (56) and tested for increased presence of each SNP in T1D versus CTR groups via logistic regression. The top enriched SNP was rs6422701, with the T allele being overrepresented in the T1D cohort (Supplemental Table 5). The T allele of rs6422701 was associated with decreased *PDCD1* mRNA expression in whole blood in GTEx (Supplemental Figure 15A). We saw significantly decreased PD-1 expression on CD4^+^ Tem and CD4^+^ Temra, but not CD4^+^ Tcm or CD8^+^ Tcm, in CTR and AAb- REL with the T allele (Supplemental Figure 15, B–E), partially mirroring observations in REL versus CTR overall (Figure 6, I–L, and Supplemental Figure 14, I–L).

### Increased HLA-DR expression on monocytes with T1D-associated HLA-DR4 genotype.

**Figure 7:**
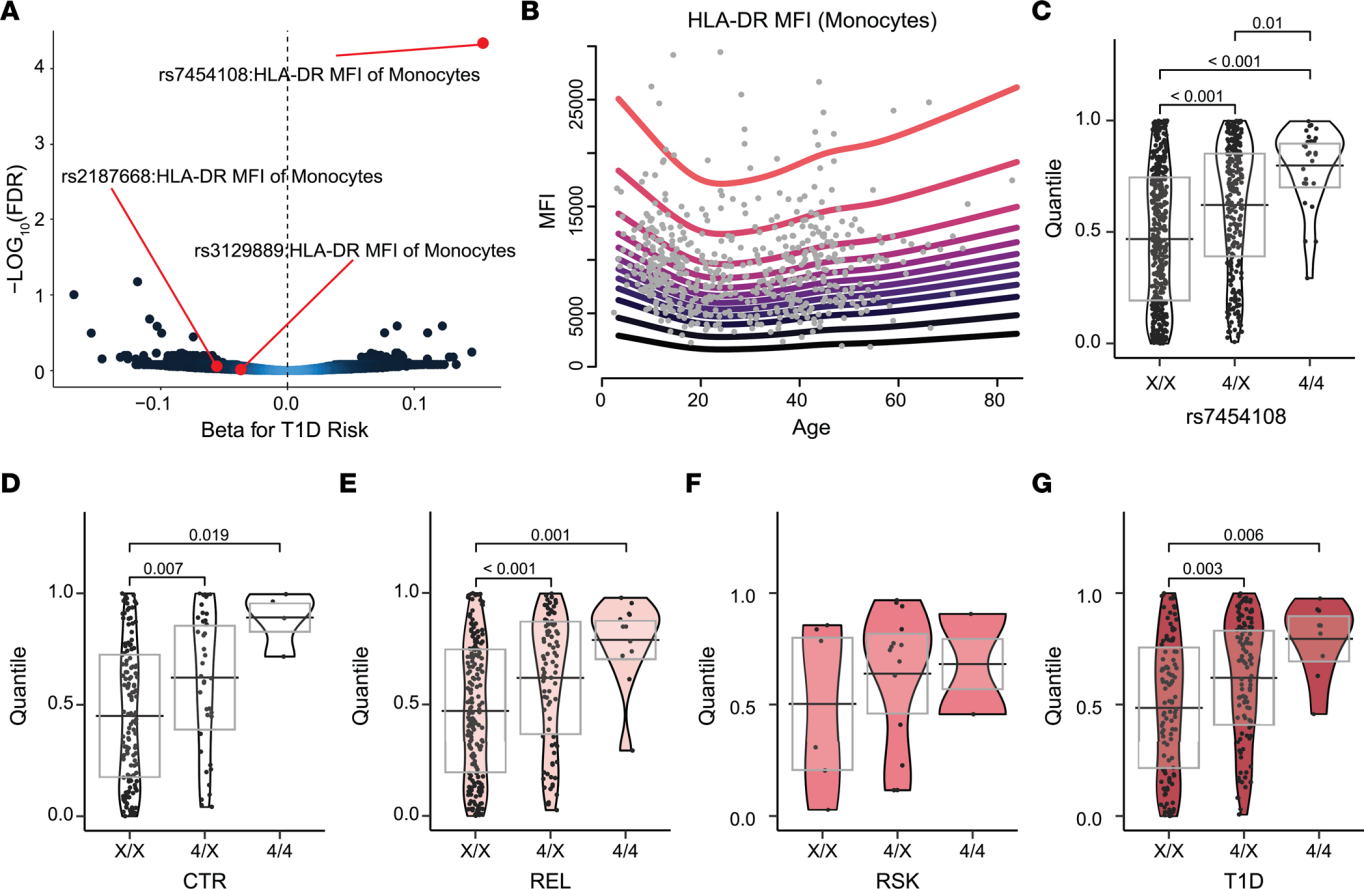
Increased HLA expression on monocytes in HLA-DR4 individuals. (**A**) Volcano plot showing QTL analysis results of all flow cytometry phenotypes versus T1D risk loci. Associations shown according to direction and effect size (β) of each SNP on T1D risk. Blue designates higher and black designates lower data density. Associations between HLA-DR MFI on monocytes and tag SNPs for HLA-DR4 (rs7454108), -DR3 (rs2187668), and -DR15 (rs3129889) T1D risk or protective class II HLA alleles highlighted in red. (**B**) The GAMLSS model fit on all AAb- (CTR and REL combined, *n* = 562) to correct for age. Quantiles of HLA-DR MFI on monocytes in (**C**) whole cohort (*n* = 806), (**D**) CTR (*n* = 248), (**E**) REL (*n* = 302), (**F**) RSK (*n* = 24), (**G**) and T1D participants (*n* = 232) according to number of copies of HLA-DR4. Significant *P* values shown on graph (Kruskal-Wallis test with post hoc Dunn’s test and Benjamini-Hochberg multiplicity adjustment). (See Supplemental Table 6.) X, any HLA-DR allele other than DR4.

While most of the immune features associated with T1D (Figure 5) were comparable between AAb- CTR and AAb- REL (Figure 3B and Figure 6, A–H), some phenotypes showed distinct differences between these groups (Figure 6, I–L). As genetic loci contributing to risk for T1D are enriched in REL (5), we performed QTL analysis on genetically unrelated participants to test for associations between age-corrected flow cytometric phenotypes and genotypes at 240 T1D risk variants (29–31) found at ≥5% minor allele frequency (MAF) in our cohort, with RSK or T1D status, sex, and population stratification as covariates. Following adjustment for multiple testing of genotypes and phenotypes, a significant association (FDR < 0.05 corrected by Benjamini-Hochberg multiplicity adjustment) was observed between the rs7454108 T1D risk genotype and increased HLA-DR MFI on monocytes (Figure 7A and Supplemental Table 6). As rs7454108 tags the high-risk HLA-DR4-DQ8 haplotype (29, 57, 58), we asked whether other HLA haplotypes carrying strong risk or protection from T1D likewise impacted this phenotype. However, we did not find evidence of association with monocyte HLA-DR MFI for the high-risk HLA-DR3-DQ2 haplotype (rs2187668; refs. 5, 29) or the dominant protective HLA-DR15-DQ6 haplotype (rs3129889; refs. 5, 29) (Figure 7A). Monocyte HLA-DR expression showed no age dependence (Figure 7B). The GAMLSS-corrected data demonstrated evidence of a genotype-dosage effect (Figure 7C). Specifically, HLA-DR MFI was increased in participants heterozygous for HLA-DR4 as compared with those carrying other HLA class II genotypes (DRX/X) and further increased in participants homozygous for HLA-DR4 (Figure 7C). Notably, the association between HLA-DR4 genotype and HLA-DR MFI on monocytes was present in all groups, suggesting that this genetic driver of immune phenotype may act independently of AAb positivity or disease status (Figure 7, D–G).

## Discussion

We conducted flow cytometric analysis of peripheral blood from a well-characterized cross-sectional cohort (n = 826), to understand how risk for autoimmune diabetes intersects with age, impacting the broad immune landscape. These data revealed striking dynamics of immune age within the adaptive compartment, which could be used to predict chronological age with a high degree of accuracy in CTR under 30 years of age. This resulting data set corroborates a list of cellular features that change dramatically as a function of age (e.g., increased CD4^+^ T cells and decreased B cells and CD8^+^ T cells, with shifts from naive to memory populations in adaptive immune cells over the life span; refs. 36–39). This complements prior descriptions of age-associated serological changes (59) and alterations in the epigenetic DNA methylation “clock” (60). Moreover, our data fill important gaps from prior studies (13, 61–63) to understand changes in the pediatric immune system — a critical period for understanding normal frequencies and cellular distributions.

Our model revealed accelerated immune aging across the first 30 years of life and numerous cellular features associated with T1D after GAMLSS age correction. Our age correction model thus enabled the assessment of phenotypic changes as influenced by T1D, overcoming a challenge of cross-sectional studies where donor age distributions are often skewed. This is of particular value to biomarker studies in pediatric diseases, where sampling of age-matched CTR is often limited. Beyond this, using our cross-sectional immunophenotyping data set, we built a model capable of predicting T1D status with internal prediction performance at 82.3% accuracy. Evaluation of this model in larger pre-T1D cohorts and longitudinal sampling will be necessary to validate its possible utility for T1D prediction and monitoring.

Considering age is a key aspect of understanding T1D pathogenesis. The first hallmark of β cell autoimmunity (i.e., emergence of multiple islet AAbs) often occurs within the first 2 years of life (6). Younger age of onset has also been associated with the highest risk HLA-DR3/4 diplotype (5), a prominent T and B cell insulitic lesion in the pancreas of T1D organ donors (9, 10), a bias toward IFN-γ:IL-10 T cell autoreactivity (64), and more acute clinical loss of endogenous C-peptide (29). While other studies have considered the impact of age on the changing immune system in T1D (65), we believe our study is unique by covering a large, multidecade age cohort, including a small group of ≥2AAb+ (i.e., stage 1–2 T1D; ref. 27) participants, and implementing a strategy to account for age differences.

In an effort to explain the observed accelerated aging, we examined T1D GRS1 (5) and clinical covariates associated with disease duration and glycemic dysregulation. Modest associations emerged with BMI percentile, disease duration, and rested blood glucose level but not with polygenic risk. Thus, we posit that accelerated immune aging observed may result from chronic inflammation, as has been described previously (66–70), and/or hyperglycemic stress, as key features of T1D. High-risk participants who progressed to disease in The Environmental Determinants of Diabetes in the Young cohort displayed chronic enteroviral shedding (71), evidence of persistent infection and inflammation. In addition, increased risk of infection in all diabetes, but also in T1D when compared with type 2 diabetes, highlights that individuals with T1D have multiple impacts on overall immune function (72).

Within this study, we replicated multiple findings in smaller cohort studies of T1D participants compared with CTR (43–45, 47–50), as well as immune aging studies (36–39), providing support for both the age prediction and T1D prediction capacity of our models. Moreover, we identified phenotypes of immune system changes with age and T1D, which to our knowledge, have not been previously reported. Our data set is readily accessible via ImmPort (accession number: SDY2299) and in an interactive format (https://ufdiabetes.shinyapps.io/ImmScape/) for public visualization and comparisons to other diseases and cohorts of interest. Phenotypic shifts associated with both aging and T1D generally reflected accelerated aging in their directionality. However, 2 age-associated phenotypes were reversed in T1D relative to CTR aging trends: naive CD8^+^ T cell and CD8^+^CD38^+^HLA-DR^–^ T cell frequencies were increased in T1D, despite decreasing with age in CTR. These phenotypes may reflect the same cell subset, considering that the majority of naive CD8^+^ T cells are CD38^+^HLA-DR^–^ (Supplemental Figure 3). Peripheral naive T cells are largely maintained through homeostatic expansion and tonic T cell receptor (TCR) signaling (73). CD8^+^ T cells usually represent a shrinking proportion of the T cell pool over time (74). Their increase in the periphery in T1D participants herein may reflect thymic output, increased expansion, and/or differentiation to stem cell memory T cells (CD45RA^+^CXCR3^+^CCR7^+^CD27^+^CD28^+^CD95^+^) (75), which could not be distinguished from naive T cells in this data set.

The observed increase in CXCR3 expression across multiple T cell subtypes could influence maturation potential, trafficking, and/or polarization to promote disease pathogenesis. Findings from murine models corroborate a role for CXCR3 in T cell trafficking to the islets and autoimmune diabetes development (76), indicating Th1 skewing as a globally dysregulated phenotype in T1D. Higher CXCR3^+^ memory T cell frequencies were shown in high-risk AAb+ REL compared with low-risk relatives (77), suggesting linkage to disease processes (77). CXCR3^+^ naive T cells are reportedly enriched in autoreactivity in mice and are thought to contain higher affinity (CD5^hi^) autoreactive TCRs (78). Additionally, expression of CXCR3 on naive CD8^+^ T cells has previously been associated with enhanced effector phenotype differentiation (79, 80).

The observed shifts in PD-1 expression in T1D extend previous findings of decreased mRNA and protein levels in CD4^+^ T cells of T1D participants (81) by defining decreased PD-1 on particular subsets (CD4^+^ Tem, CD4^+^ Temra, CD4^+^ Tcm, and CD8^+^ Tcm). These data indicate that the PD-1 pathway may serve as a critical negative checkpoint for maintaining tolerance to islet β cells (82). Observing reduced PD-1 MFI within REL suggests a potential genetic predisposition toward impaired PD-1 expression and should be subjected to validation in larger cohorts. Our finding regarding monocyte HLA-DR overexpression in HLA-DR4 participants builds upon the prior observation of this association in cord blood (83). Increased activation and/or antigen presentation afforded by the increased expression could theoretically alter aspects of T cell selection and cellular differentiation. These phenotypic observations regarding expression of CXCR3, PD-1, and HLA-DR warrant future targeted studies on the mechanism and downstream impact of the observed altered expression levels.

In T1D, we noted amplification of immune aging trajectories but do not have sufficient data in very young participants (e.g., birth to age 7) to understand when this trend is initiated and did not assess longitudinal samples to compare to preclinical status. As result of this data sparsity at either end of the life span, our interpretation of findings applies most directly to the impact of diagnosed disease on immunophenotypes after age correction of data, particularly in individuals younger than 30 years. The plateau in model performance over age 30 may result from lack of other indicators of overall aging and inflammation outside the scope of this study. Given the variable duration of disease in our cohort, association of immune phenotypes with progression of T1D in RSK individuals (≥2AAb+) would be best studied in longitudinal samples. Potential covariates of interest include pubertal status and time of blood sample draw (84), which were not recorded or included in the analysis. Beyond the immunophenotypes and effector molecules investigated herein, other immunological compartments of potential interest include mast cells, eosinophils, and basophils (85, 86), along with tissue-resident populations (11, 87–89). Experiments to address these questions are ongoing.

Our efforts to identify a signature of immune system age, as well as cellular phenotypes associated with T1D, provide a number of additional targets for consideration in precision medicine–based therapies (e.g., CXCR3^+^ T cells, the PD-1 costimulatory axis, antigen presentation on monocytes, specifically in individuals with HLA-DR4). Moreover, these results provide cellular and pathway targets for consideration in mechanistic studies of prior trials where efficacy differed according to participant age (e.g., rituximab [anti-CD20, TN05], low-dose anti–thymocyte globulin [ATG, TN19], abatacept [CTLA4-Ig, TN09 and TN18]) (90, 91) and in future intervention studies. Importantly, immune-directed therapeutic interventions aimed at interrupting the autoimmune destruction of β cells, at or prior to clinical diagnosis of T1D, often have outcomes impacted by the age of participants at the time of drug treatment (reviewed in ref. 92). We suggest this observation reflects altered immune system constituents that shape therapeutic response (or lack thereof), depending on the target and drug mechanism of action. The approach of considering immune age, phenotype, and chronological age of trial participants may improve clinical response profiles and progress toward precision medicine–based strategies to prevent and reverse T1D.

## Methods

### Study design 

Individuals were recruited from the general population and outpatient endocrinology clinics at the University of Florida (UF; Gainesville, Florida, USA), Nemours Children’s Hospital (Orlando, Florida, USA), and Emory University (Atlanta, Georgia, USA). Following procurement of written informed consent, peripheral blood samples were collected into the UF Diabetes Institute (UFDI) Study Bank from 826 nonfasted participants by venipuncture. Samples were collected in EDTA-coated vacutainer tubes for flow cytometry, CBC, HbA1c, TCRβ-sequencing, and genotyping assays; serum separator vacutainer tubes for islet AAb and CMV IgG antibody measurement; and sodium fluoride/potassium oxalate–coated tubes (BD Biosciences) for rested blood glucose quantification. Samples were shipped or rested overnight in order to standardize duration between collection and evaluation at UF (33). Data were collected from all incoming blood samples to the UFDI from 2014–2018, hence the lack of age-matching between clinical subgroups based on clinical status. Importantly, participants had no reported infection or malignancy at time of blood draw, and sample collection preceded the COVID-19 pandemic. Demographic and clinical information are presented in Table 1.

### AAb measurement

Islet Autoantibody Standardization Program–evaluated (IASP-evaluated) ELISAs (93) were performed on serum to measure T1D-associated AAbs reactive to glutamic acid decarboxylase 65, insulinoma-associated protein-2, and zinc transporter-8, which respectively performed with AUROCs of 0.936, 0.876, and 0.917 in the most recent IASP workshop. REL and CTR participants were considered RSK if possessing reactivity to at least 2 of the screened AAb specificities (27).

### Flow cytometry

Rested whole blood samples were stained with 6 flow cytometry panels, as we have previously described (18, 32). Briefly, 200 μL of whole blood was incubated with antibodies (Supplemental Table 7) for 30 minutes at room temperature. Red blood cells were lysed using 1-step Fix/Lyse Solution (eBioscience) and cells washed with staining buffer. Data were acquired on an LSRFortessa (BD Biosciences) within 24 hours of staining. Analyses were performed in FlowJo software (v9 and v10; BD Biosciences) with gating strategies in Supplemental Figures 1–6.

### CBC, HbA1c, and blood glucose measurement

Rested whole blood samples were characterized using the Coulter Ac•T 5diff CP (Cap Pierce) Hematology Analyzer (Supplemental Table 1). HbA1c was measured with the DCA Vantage Analyzer (Siemens) and rested blood glucose with the Contour Next EZ glucometer (Bayer).

### CMV status

A random subset (40.56%) of the total flow cytometry cohort was assayed for evidence of prior or primary CMV exposure by predicting serostatus from *TRBV* (TCRβ) sequences (Adaptive Biotechnologies) derived from PBMCs (94, 95). CMV status was inferred using methods and trained model described by Emerson et al. (96). Upon testing, predictions over 0.5 were deemed CMV positive. Serostatus from a CMV IgG antibody ELISA (Zeus Scientific) was available for 71.04% of all samples that were TCRβ-sequenced from our cohort. We noted reliable concordance between the 2 methods of CMV status classification, as evidenced by AUROC of 0.886. In cases of discrepancy (14.83% of participants with data from both assays), CMV classification from ELISA superseded the TCRβ data as the result used for further analysis.

### Genotyping of T1D risk loci and quantitative trait loci analysis

Samples were genotyped using our custom UFDIchip SNP array (97, 98), which includes the Axiom Precision Medicine Research Array (Thermo Fisher Scientific), all content from the ImmunoChip.v2.0 (99), and previously reported credible T1D risk variants (55). QC steps and QTL assessment were performed with plink 1.9. T1D GRS1 was calculated as previously established (97).

QC measures were performed prior to association testing, as previously described (100). Participants were excluded from analysis if any of the following conditions applied: >2% of directly genotyped SNPs were missing, genetically imputed sex did not match reported sex, or heterozygosity rate differed ±3 SD from the mean of all samples (2.91% of participants excluded based on these criteria). Identity by descent calculations were used to remove related individuals with pi-hat > 0.2, randomly retaining 1 participant in each related pair (101), resulting in the exclusion of 40.56% of participants. Genotypes at 277 previously curated T1D risk loci (29–31) were pulled directly from the UFDIchip or, if missing from the chip, obtained from imputation to 1000 Genomes Phase 3 (v5) or Human Reference Consortium (vr1.1) using the Michigan Imputation Server (102), with imputed genotypes from the reference cohort providing the higher imputation quality used for QTL analysis (mean *R*^2^ = 0.952). SNPs that were missing in >2% of participants, with a MAF < 5%, or failing Hardy-Weinberg equilibrium at *P* < 1 × 10^–6^ were excluded. A linear regression analysis was performed using PLINK (103) for genotype association with GAMLSS-corrected flow cytometric phenotypes, with clinical group (CTR or REL, RSK, T1D) and sex included as categorical covariates and 10 multidimensional scaling components as continuous covariates to account for population stratification. *P* values were adjusted for multiple testing of genotypes and phenotypes to generate an FDR using the Benjamini-Hochberg method with R/p.adjust. A volcano plot was created using GraphPad Prism v7.0 depicting –log_10_(FDR) and the magnitude of the association between the phenotype and the T1D risk allele (β coefficient) (29–31).

### Statistics

#### Phenotype trajectories.

Data are presented as mean ± SD, and all tests were 2 sided unless otherwise specified. Technical and biological CV in the flow cytometric assay were assessed on a cohort of 12 samples that were run in technical duplicates using GraphPad Prism software version 7.0. Spearman’s correlations between immune phenotypes and age were computed using R/pspearman.

For characterizing the flow cytometric phenotype dynamics over age in CTR and REL, missing phenotype data due to failure of visual QC validation of staining (<5.03% of data per phenotype) were median-imputed. Smoothing splines of each phenotype versus age were fit with 3 degrees of freedom using the smooth.spline R function (104).

To model the dynamics of each phenotype across age, we log-transformed the imputed data, adding a constant equal to 1 or, for phenotypes with possible values less than 1, we added an additional constant to shift all values larger than or equal to 0. We then *z*-transform scaled each phenotype prior to fitting a smoothing spline, as described above, across age with 3 degrees of freedom. The smoothed trajectory predictions were restricted to an age range of 5–75 years to avoid predicting outside the observed age range of our other cohorts present. The phenotype trajectories were then clustered using hierarchical clustering with the complete method and the Canberra distance metric to group phenotypes with similar trajectory patterns over age. Heatmaps of processed data from AAb- CTR and REL samples, ordered by participant age on the *x* axis and dendrogram clustering on the *y* axis, were created using R/gplots. R/ggplot2 and R/ggpubr were used to create figures of overlaid smooth splines of phenotypes for 4 clusters. The same procedures were applied to obtain T1D phenotype trajectories, and the T1D smoothed trajectories were plotted keeping the same *y* axis order for comparison with age-related trajectories in CTR and REL.

#### Comparing smooth trajectory fits.

To compare the spline fits for the 2 cohorts (CTR and REL combined vs. T1D), we computed the trajectory shift as the difference in the overall average trajectory value between cohorts. A 2-tailed *t* test was used to test for significant shifts for each of the 4 clusters. To determine the initial trajectory direction, for each phenotype in each cohort, we calculated the mean value of successive differences in the trajectory over years 5–15, with a positive value indicating an increasing trajectory and negative value a decreasing trajectory. We estimated the number of times the trajectories crossed by examining the number of sign changes along the successive differences across the entire age range obtained.

#### Immunophenotype age model.

We used the random lasso (35) method on CTR to identify features associated with immune aging. We first imputed missing values within each feature to its median and then *z*-transform scaled. With chronological age as the response and all features as predictors, we repeated our random lasso procedure on 1,000 random train-test subsets with 80% of the individuals in a training set and the remaining 20% as a held-out test set. The first stage of the random lasso consisted of 1,000 bootstrap samples of the training set, with each bootstrap estimating the coefficients on 15%–20% randomly selected features. The initial variable importance score for all phenotypes was calculated as the average coefficient value. The second stage of the random lasso used another 1,000 bootstrap samples from the training set, and 10% of the features were fit in a lasso model using the initial importance score as the selection probability. For each data split, the overall variable importance score was obtained by averaging the bootstrapped coefficients. A final variable importance score represented the average of 1,000 random data splits.

Of the 192 features evaluated, 182 had non-zero average coefficients from the random lasso procedure; thus, we further implemented a procedure to obtain an importance score cutoff. All variables higher than our cutoff threshold were considered predictive of age. To determine the optimal cutoff value, we ran 5-fold cross-validation and evaluated the root mean squared error (RMSE) for each cutoff in the random lasso procedure. We estimated an elbow point per fold to minimize RMSE. We repeated this 5-fold cross-validation 20 times, and the mean of elbow points was the final cutoff. The final linear model is the averaged coefficients from the random lasso models, which we then applied to the CTR, REL, and T1D cohorts to predict immunological age.

To internally validate our age prediction model, we similarly trained a random lasso model on additional cohorts: 1) combined CTR and REL, 2) CTR younger than 30, and 3) CTR older than 30.

#### Age prediction within T1D and control participants.

A linear regression model of predicted age was fit on the chronological age and a factor for clinical group (T1D versus combined CTR and REL). We then fit a multivariable model of predicted age on chronological age, sex, ethnicity, reported race, BMI percentile calculated as previously described (105), HbA1c, rested blood glucose, and GRS1. Continuous variables were *z*-transform scaled to obtain standardized coefficients. To ensure a sufficient number of individuals were represented across groups, the model was limited to participants with reported race of African American or Caucasian (Table 1).

Within the T1D cohort, we again fit a multivariable model for the variables described above in addition to disease duration and diagnosis age. Due to the correspondence in our cohort between diagnosis age and age at sampling time, Δage between chronological and predicted age was not used as the outcome variable in this analysis. Instead, we used residuals determined from a linear regression of predicted age and chronological age to explain factors associating with differences between chronological and predicted age in T1D. The standardized multivariable model was fit to the CTR and REL cohorts separately.

#### Adjusting for age using GAMLSS model.

In order to obtain age-adjusted centiles of immunophenotypes in CTR and T1D cohorts, we employed a weighted GAMLSS (41, 42). In CTR and REL, each phenotype was modeled as a cubic spline function of age using either a Box-Cox-t (BCT) distribution or normal (NO) distribution, depending upon the skewness of the phenotype distribution. A skewness cutoff of 0.5 was determined empirically, with distributions having skew larger than 0.5 fit using the BCT distribution. For phenotypes using the BCT distribution, values were shifted by a constant value to ensure positivity as necessary. Weights were assigned according to the age distribution density such that ages younger than 10 years were assigned a relative weight of 10, ages older than 70 years were assigned a relative weight of 0.1, with all other ages assigned a weight of 1. We then used the distribution function (pBCT or pNO in the GAMLSS R package) and the predicted parameter values from the weighted GAMLSS model to obtain age-corrected quantile values for all individuals. We note 2 exceptions to the above: 1) memory Treg CD25 index had a skewness of 0.54 but was better fit using the normal distribution, and 2) CXCR3^hi^ (CD8 Temra) was fit using the unweighted GAMLSS model (all weights equal to 1) because of errors using the weighted model.

#### Identifying T1D-associated immunophenotypes.

The age-corrected quantile values were used to compare T1D versus RSK, REL, and CTR individuals. A nonparametric Kruskal-Wallis test was performed for each phenotype followed by a post hoc Dunn’s test with a Benjamini-Hochberg multiplicity adjustment. Multiplicity-adjusted *P* < 0.05 was considered significant.

#### Immunophenotype prediction model.

We first imputed missing values within each phenotype to its median and then *z*-transform scaled each phenotype. To handle correlations and dependencies in the phenotypes, we used principal component analysis. The first 30 components were selected based on an elbow plot of explained variance and used as predictors in generalized linear model of T1D versus CTR status. The AUROC was averaged over 1,000 independent samplings of an 80:20 train-test set split.

#### Comparison to IMM-AGE score.

We applied the same approach described in Alpert et al. (26) to estimate an immune pseudotime using the diffusion maps algorithm. Specifically, we used the default options in the DiffusionMap function in the R/destiny package on our phenotype matrix.

### Study approval

Participants provided written informed consent prior to study enrollment and sample collection, in accordance with IRB-approved protocols at the UF, Nemours Children’s Hospital, and Emory University.

### Data availability

Flow cytometric data are available at ImmPort (https://www.immport.org, accession number: SDY2299). Supporting Data Values associated with this manuscript are provided in the supplement. Data are also available for visualization and analysis via an interactive R/Shiny application (ImmScape; https://ufdiabetes.shinyapps.io/ImmScape/) and from the corresponding author upon reasonable request.

## Author contributions

DJP, DAS, MAA, and TMB conceived the study. DJP, MRS, XD, MAB, and RB developed methodology. MRS, XD, and RB performed formal analysis. DJP, PT, JMM, LDP, and KM investigated. TMB, MAA, AM, LMJ, MJH, and DAS provided resources. MRS, XD, DJP, and RSM performed data curation. DJP, RB, XD, MRS, and PT visualized data. TMB, MAA, CEM, MRS, PT, and LDP acquired funding. DJP, MRS, RB, and TMB were project administrators. DJP, RB, MAB, and TMB provided supervision. MRS, MAB, XD, DJP, and RB wrote the original draft. MRS, XD, DJP, JMM, PT, ALP, LDP, KM, RSM, MAB, AM, PC, LMJ, CEM, CHW, MJH, DAS, MAA, RB, and TMB reviewed and edited the manuscript. Authorship order among the 3 co–first authors was determined as follows: 1) MRS led manuscript writing along with data analysis and interpretation, 2) XD led formal analysis and visualization of the data, and 3) DJP developed and validated the flow cytometry staining panels, generated the data, and performed preliminary analyses.

## Supplementary Material

Supplemental data

Supporting data values

## Figures and Tables

**Table 1:**
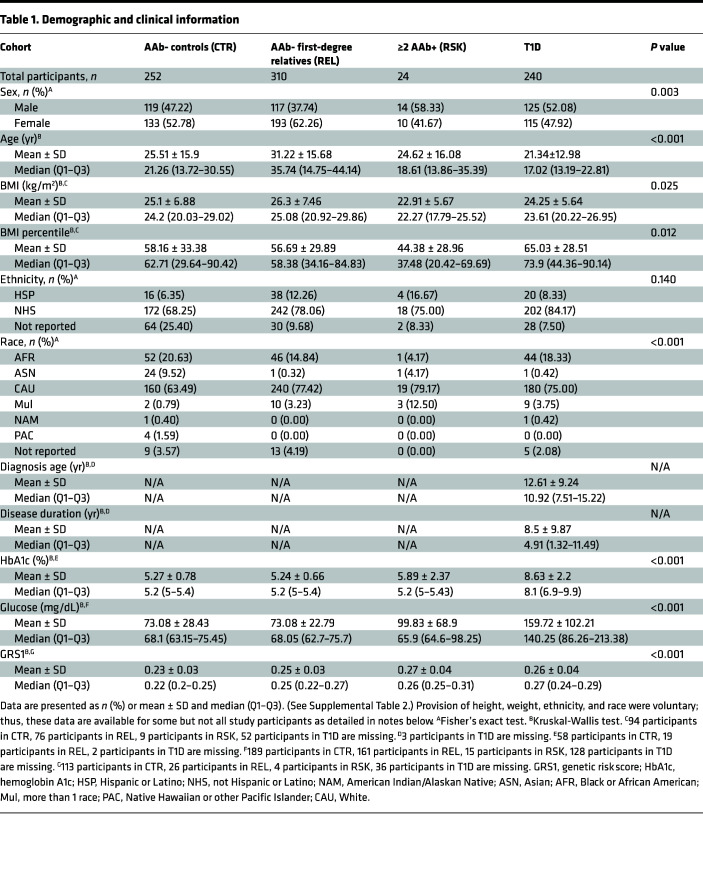
Demographic and clinical information

## Version 1 07/27/2023

In-Press Preview

## Version 2 09/08/2023

Electronic publication
