# Supplementary material for: Human immune phenotyping reveals accelerated aging in type 1 diabetes
Source: JCI Insight. 2023 Sep 8;8(17):e170767. doi: 10.1172/jci.insight.170767 (PMC10544250; doi:10.1172/jci.insight.170767)
Supplement: Supplemental data [file jciinsight-8-170767-s070.pdf]

Supplementary Materials

Table S1. Proportions of CBC data falling outside of established 5th-95th percentile reference ranges. Percentage of autoantibody-negative (AAb-) control (CTR), AAb- first degree relative (REL), ≥2 AAb+ (RSK), and type 1 diabetes (T1D) participants with data below (Low, <5th percentile), within (Normal, 5th-95th percentile), and above (High, >95th percentile) reference ranges reported for the instrument (Ac*T 5diff Cap Pierce Hematology Analyzer, Beckman Coulter). Data were analyzed by Chi-square tests with Bonferroni adjustment with significant differences, defined as adjusted p<0.05, shown in bold. (See also Figure 1)

**CBC Phenotype**	**CTR (%)**				**REL (%)**				**RSK (%)**				**T1D (%)**			
	**Low**	**Normal**	**High**	**Adj. p**	**Low**	**Normal**	**High**	**Adj. p**	**Low**	**Normal**	**High**	**Adj. p**	**Low**	**Normal**	**High**	**Adj. p**
WBC (10^3/µL)	6.2	87.1	6.7	1.000	2.4	89.7	7.9	0.950	4.5	90.9	4.5	1.000	4.2	89.7	6.1	1.000
RBC (10^6/µL)	4.1	89.2	6.7	1.000	2.1	94.8	3.1	1.000	0.0	95.5	4.5	1.000	3.7	88.4	7.9	1.000
HGB (g/dL)	6.7	89.2	4.1	1.000	6.9	90.3	2.8	1.000	0.0	90.9	9.1	1.000	4.7	88.4	7.0	1.000
HCT (%)	6.2	86.1	7.7	1.000	4.5	85.9	9.7	0.105	0.0	81.8	18.2	0.930	3.3	84.2	12.6	**<0.001**
MCV (fL)	6.7	83.5	9.8	0.335	4.5	86.6	9.0	0.639	0.0	90.9	9.1	1.000	1.4	92.6	6.0	1.000
MCH (pg)	8.2	87.1	4.6	1.000	6.2	88.6	5.2	1.000	0.0	95.5	4.5	1.000	2.3	94.4	3.3	1.000
MCHC (g/dL)	25.3	72.7	2.1	**<0.001**	14.1	85.9	0.0	**<0.001**	18.2	81.8	0.0	0.930	14.0	85.6	0.5	**<0.001**
RDW (%)	5.7	90.2	4.1	1.000	5.2	90.3	4.5	1.000	13.6	86.4	0.0	1.000	16.7	80.9	2.3	**<0.001**
PLT (10^3/µL)	5.7	86.6	7.7	1.000	2.1	90.0	7.9	0.548	4.5	90.9	4.5	1.000	2.3	86.0	11.6	**0.001**
MPV (fL)	1.5	95.9	2.6	1.000	1.7	94.8	3.4	1.000	0.0	90.9	9.1	1.000	1.4	94.4	4.2	1.000
NE (%)	18.6	78.4	3.1	**<0.001**	15.5	78.3	6.2	**<0.001**	9.1	90.9	0.0	1.000	18.6	75.3	6.0	**<0.001**
LY (%)	4.6	77.3	18.0	**<0.001**	7.2	82.1	10.7	**<0.001**	0.0	95.5	4.5	1.000	8.4	76.7	14.9	**<0.001**
MO (%)	1.0	92.3	6.7	1.000	3.1	89.3	7.6	1.000	0.0	90.9	9.1	1.000	0.5	93.5	6.0	0.657
EO (%)	8.8	90.7	0.5	0.103	8.6	90.7	0.7	**0.008**	4.5	86.4	9.1	1.000	11.6	87.0	1.4	**<0.001**
BA (%)	1.0	94.8	4.1	1.000	0.7	93.8	5.5	0.268	0.0	100.0	0.0	1.000	0.0	94.4	5.6	0.273
NE# (10^3/µL)	11.3	82.5	6.2	0.014	7.2	84.1	8.6	0.239	4.5	90.9	4.5	1.000	12.6	80.5	7.0	**<0.001**
LY# (10^3/µL)	3.1	83.0	13.9	**<0.001**	2.8	88.3	9.0	0.186	0.0	100.0	0.0	1.000	0.9	89.3	9.8	**0.017**
MO# (10^3/µL)	0.0	82.5	17.5	**<0.001**	2.1	77.9	20.0	**<0.001**	0.0	95.5	4.5	1.000	1.4	84.2	14.4	**<0.001**
EO# (10^3/µL)	13.9	84.5	1.5	**<0.001**	12.1	86.9	1.0	**<0.001**	18.2	77.3	4.5	1.000	12.6	86.5	0.9	**<0.001**
BA# (10^3/µL)	0.5	91.2	8.2	0.206	0.3	91.4	8.3	**0.006**	0.0	100.0	0.0	1.000	0.0	93.0	7.0	0.143

Table S2. Demographic and clinical information for individuals aged <30 years. Data are presented as n (percentage, %) or mean ± SD and Median IQR (Q1-Q3).

Cohort	AAb- Controls (CTR)	AAb- First-Degree Relatives (REL)	≥2 AAb+ (RSK)	T1D	P value
Total Subjects, n	187	121	17	193	
Sex, n (%)[†]					0.405
Male	90 (48.13)	57 (47.11)	11 (64.71)	103 (53.37)	
Female	97 (51.87)	64 (52.89)	6 (35.29)	90 (46.63)	
Age (years)[‡]					<0.001
Mean ± SD	17.71±6.62	13.33±5.05	15.42±5.72	15.59±4.61	
Median (Q1-Q3)	18 (11.87-23.1)	12.9 (10.14-16.4)	14.32 (10.1-19)	15.24 (12.21-18.49)	
BMI (kg/m2)[‡A]					0.016
Mean ± SD	24.14±7.1	21.62±4.9	21.9±5.82	23.2±4.72	
Median (Q1-Q3)	22.49 (19.52-27.17)	20.64 (18.61-24.03)	20.88 (17.47-23.78)	22.84 (19.78-25.92)	
BMI percentile[‡A]					0.169
Mean ± SD	62.9±34.02	65.5±30.09	45.14±31.36	68.23±26.89	
Median (Q1-Q3)	71.24 (31.4-95.2)	75.4 (42.55-90.92)	37.58 (17.77-76.57)	77.5 (50.05-90.95)	
Ethnicity, n (%)[†]					0.123
HSP	11 (5.88)	17 (14.05)	3 (17.65)	15 (7.77)	
NHS	128 (68.45)	96 (79.34)	13 (76.47)	161 (83.42)	
Not Reported	48 (25.67)	8 (6.61)	1 (5.88)	17 (8.81)	
Race, n (%)[†]					<0.001
AFR	38 (20.32)	25 (20.66)	1 (5.88)	41 (21.24)	
ASN	20 (10.7)	0 (0)	1 (5.88)	1 (0.52)	
CAU	121 (64.71)	86 (71.07)	12 (70.59)	136 (70.47)	
Mul	1 (0.53)	6 (4.96)	3 (17.65)	9 (4.66)	
NAM	0 (0)	0 (0)	0 (0)	1 (0.52)	
PAC	3 (1.6)	0 (0)	0 (0)	0 (0)	
Not Reported	4 (2.14)	4 (3.31)	0 (0)	5 (2.59)	
Diagnosis Age[‡B]					N/A
Mean ± SD	N/A	N/A	N/A	10.45±4.64	
Median (Q1-Q3)	N/A	N/A	N/A	10.29 (7.39-13.47)	
Disease Duration (years) [‡B]					N/A
Mean ± SD	N/A	N/A	N/A	5.18±4.83	
Median (Q1-Q3)	N/A	N/A	N/A	3.44 (1.12-8.96)	

HbA1c (%)[‡C]					<0.001
Mean ± SD	5.24±0.62	5.14±0.6	5.16±0.26	8.96±2.21	
Median (Q1-Q3)	5.2 (5-5.4)	5.1 (4.9-5.27)	5.2 (5-5.3)	8.6 (7.3-10.2)	
Glucose (mg/dL)[‡D]					<0.001
Mean ± SD	71.65±33.05	72.53±24.69	72.57±14.29	164.35±108.6	
Median (Q1-Q3)	67.25 (62.6-71.42)	66.5 (62.05-74.1)	65.55 (64.75-76.89)	143 (87.15-228)	
GRS1[‡E]					<0.001
Mean ± SD	0.23±0.03	0.25±0.03	0.28±0.04	0.26±0.04	
Median (Q1-Q3)	0.22 (0.2-0.24)	0.25 (0.22-0.27)	0.28 (0.24-0.31)	0.27 (0.24-0.29)	

*Provision of height and weight was voluntary; thus, these data are available for some, but not all study subjects.

[†]Fisher’s exact test, [‡]Kruskal Wallis test

[A]70 participants in CTR, 23 participants in REL, 5 participants in RSK, 37 participants in T1D are missing.

[B]1 participants in T1D are missing.

[C]42 participants in CTR, 7 participants in REL, 1 participants in T1D are missing.

[D]148 participants in CTR, 62 participants in REL, 11 participants in RSK, 104 participants in T1D are missing.

[E]95 participants in CTR, 16 participants in REL, 3 participants in RSK, 29 participants in T1D are missing.

Abbreviations: HSP, Hispanic or Latino; NHS, Not Hispanic or Latino; None, Unknown or Not Reported; NAM, American Indian/Alaskan Native; ASN, Asian; AFR, Black or African American; Mul, More Than One Race; PAC, Native Hawaiian or Other Pacific Islander; None, Unknown or Not Reported; CAU, White.

Table S3. Multivariable model of disease-relevant features with residual age in T1D, CTR and REL individuals. Standardized regression estimates and standard errors are shown for continuous covariates, and the Type II ANOVA p-values are shown for categorical covariates with p-values <0.05 shown in bold. (See also Figure 3C).

**T1D**			
**Disease-Relevant Features**	**Estimate**	**Std. Error**	**p-value**
**Continuous features**			
T1D Diagnosis Age	0.878	0.559	0.120
T1D Duration	1.470	0.483	**0.003**
BMI %tile	1.752	0.468	**<0.001**
HbA1C	0.495	0.475	0.301
Rested Blood Glucose	1.102	0.494	**0.029**
GRS1	-0.236	0.540	0.663
**Categorical features**			
Sex	–	–	0.228
Ethnicity	–	–	0.606
Race	–	–	0.957

**CTR**			
**Disease-Relevant Features**	**Estimate**	**Std. Error**	**p-value**
**Continuous features**			
BMI %tile	1.500	1.159	0.215
HbA1C	-0.202	1.510	0.895
Rested Blood Glucose	0.438	1.818	0.813
GRS1	-0.279	1.088	0.801
**Categorical features**			
Sex	–	–	0.813
Ethnicity	–	–	0.936
Race	–	–	0.990

**REL**			
**Disease-Relevant Features**	**Estimate**	**Std. Error**	**p-value**
**Continuous features**			
BMI %tile	0.299	0.500	0.554
HbA1C	1.032	0.782	0.194
Rested Blood Glucose	-0.007	1.276	0.995
GRS1	0.038	0.538	0.944
**Categorical features**			
Sex	–	–	0.360
Ethnicity	–	–	0.427
Race	–	–	0.667

Table S4. Cellular subset frequencies and phenotypes assessed in peripheral blood of T1D and controls. Outcome measures from flow cytometry (gated cell population frequency, mean fluorescence intensity (mfi), or normalized marker intensity (index) of (indicated parent population)) or complete blood count (CBC) assessment were compared in T1D and control subjects. Testing was performed using a non-parametric Kruskal Wallis followed by a post-hoc Dunn's test with a Benjamini-Hochberg multiplicity adjustment. (See also Figure 4C)

Cell frequency or phenotype (Parent population)	Definition	Difference	Mean Quantile Difference	p-value
CXCR3^{lo} (CD8 Naïve)	CD183^{lo} % of CD3^{+}CD4^{-} Naive	T1D ↑	0.21	<0.001
CXCR3^{lo} (CD8 CD45RO^{-})	CD183^{lo} % of CD3^{+}CD4^{-}CD45RO^{-}	T1D ↑	0.18	<0.001
CXCR3^{+} (Naive CD8)	CD183^{+} % of Naive CD8	T1D ↑	0.142	<0.001
Naive (CD8)	Naive % of CD8	T1D ↑	0.113	<0.001
Hemoglobin	HGB (g/dL)	T1D ↑	0.112	0.001
CD123^{+} (MNCs)	CD123^{+} % of MNCs [mononuclear cells]	T1D ↑	0.11	<0.001
Hematocrit	HCT (%)	T1D ↑	0.108	0.003
PD1^{+} (Naive CD4)	CD279^{+} % of Naive CD4	T1D ↑	0.107	<0.001
CXCR3^{lo} (CD8 Temra)	CD183^{lo} % of CD3^{+}CD4^{-} Temra	T1D ↑	0.101	0.001
Activated Memory (Treg)	CD45RO^{+}HLADR^{+}CCR4^{+} % of Treg	T1D ↑	0.098	0.002
Memory Th2 (Tconv)	CD45RO^{+}CCR4^{+} % of Tconv	T1D ↑	0.096	0.003
MCV	MCV [mean corpuscular volume] (fL)	T1D ↑	0.092	0.009
Granulocyte (Leukocytes)	Granulocyte % of Leukocytes	T1D ↑	0.09	0.002
MCH	MCH (pg)	T1D ↑	0.089	0.011
PD1^{+} (CD8 Temra)	CD279^{+} % of CD8 Temra	T1D ↑	0.089	0.009
HLA-DR mfi (Monocytes)	HLA-DR mfi of Monocytes	T1D ↑	0.088	0.014
CXCR3^{+} (Tfh)	CD183^{+} % of Tfh	T1D ↑	0.087	0.002
PD1^{+} (Naive CD8)	CD279^{+} % of Naive CD8	T1D ↑	0.085	0.009
CD38^{+}HLADR^{-} (CD8)	CD38^{+}HLADR^{-} % of CD8	T1D ↑	0.084	0.01
HLADR^{+}CD38^{+} (Memory Th17)	HLADR^{+}CD38^{+} % of CD4^{+}CD45RO^{+}CD183^{-}CD196^{+}	T1D ↑	0.078	0.024
Absolute platelet count	PLT (10^3/µL)	T1D ↑	0.076	0.019
Tcm (CD4)	Tcm % of CD4	T1D ↑	0.074	0.027
CXCR5^{+} (Naive CD4)	CD185^{+} % of Naive CD4	T1D ↑	0.073	0.028
Memory (Treg)	CD45RO^{+} % of Treg	T1D ↑	0.071	0.034
CD45RO^{+}CCR4^{+} (CD8)	CD45RO^{+}CCR4^{+} % of CD3^{+}CD4^{-}	T1D ↑	0.069	0.016
CD56^{bright} (NK)	CD56^{bright} % of NK	T1D ↑	0.067	0.03
PD1 index (Tcm CD4)	Tcm CD4 CD279mfi Rel. Diff.	T1D ↓	0.054	0.041

NK (Lymphocytes)	NK % of Lymphocytes	T1D ↓	0.064	0.048
CD38^{-}HLADR^{-} (Naive CD8)	CD38^{-}HLADR^{-} % of Naive CD8	T1D ↓	0.066	0.023
CD56dim (NK)	CD56dim % of NK	T1D ↓	0.069	0.028
PD1 index (Tem CD4)	Tem CD4 CD279mfi Rel. Diff.	T1D ↓	0.07	0.035
Lymphocytes (CBC)	LY (%)	T1D ↓	0.071	0.036
MNC (Leukocytes)	MNC % of Leukocytes	T1D ↓	0.073	0.021
CD38^{+}HLADR^{-} (CD8 Temra)	CD38^{+}HLADR^{-} % of CD8 Temra	T1D ↓	0.075	0.023
CD38^{-}HLADR^{-} (Naive CD4)	CD38^{-}HLADR^{-} % of Naive CD4	T1D ↓	0.076	0.022
CXCR3^{hi} (CD8 CD45RO^{-})	CD183^{hi} % of CD3^{+}CD4^{-}CD45RO^{-}	T1D ↓	0.08	0.007
Transitional (B cells)	Transitional % of B cells	T1D ↓	0.09	0.005
PD1mfi (Temra CD4)	Temra CD4 CD279mfi	T1D ↓	0.091	0.004
PD1mfi (Tcm CD4)	Tcm CD4 CD279mfi	T1D ↓	0.092	0.001
CD38^{-}HLADR^{-} (CD8)	CD38^{-}HLADR^{-} % of CD8	T1D ↓	0.093	0.004
DN (T cells)	DN % of T cells	T1D ↓	0.106	0.001
PD1mfi (Tcm CD8)	Tcm CD8 CD279mfi	T1D ↓	0.108	<0.001
CXCR3^{-} (CD8 Temra)	CD183^{-} % of CD3^{+}CD4^{-} Temra	T1D ↓	0.108	<0.001
Tem (CD8)	Tem % of CD8	T1D ↓	0.112	<0.001
PD1mfi (Tem CD4)	Tem CD4 CD279mfi	T1D ↓	0.117	<0.001
RDW	RDW (%)	T1D ↓	0.154	<0.001
CXCR3^{-} (CD8 CD45RO^{-})	CD183^{-} % of CD3^{+}CD4^{-}CD45RO^{-}	T1D ↓	0.163	<0.001
CXCR3^{-} (CD8 Naïve)	CD183^{-} %of CD3^{+}CD4^{-} Naive	T1D ↓	0.189	<0.001
Naive (B cells)	Naive % of B cells	T1D ↑	0.066	ns
Myeloid (DCs)	Myeloid % of DCs	T1D ↑	0.065	ns
HLA-DR mfi (DCs)	HLA-DR mfi of DCs	T1D ↑	0.065	ns
CXCR5^{+} (CD8 Temra)	CD185^{+} % of CD8 Temra	T1D ↑	0.064	ns
Neutrophils (CBC)	NE (%)	T1D ↑	0.062	ns
CXCR3^{+} (CD4 Tcm)	CD183^{+} % of CD4 Tcm	T1D ↑	0.06	ns
CD38^{+}HLADR^{-} (Naive CD4)	CD38^{+}HLADR^{-} % of Naive CD4	T1D ↑	0.06	ns
CD38^{+}HLADR^{-} (Naive CD8)	CD38^{+}HLADR^{-} % of Naive CD8	T1D ↑	0.058	ns
CXCR3^{+} (CD8 Temra)	CD183^{+} % of CD8 Temra	T1D ↑	0.055	ns
CD38^{-}HLADR^{+} (CD8 Temra)	CD38^{-}HLADR^{+} % of CD8 Temra	T1D ↑	0.054	ns
RBC count	RBC (10^{6}/µL)	T1D ↑	0.054	ns
Activated Memory (CD8)	CD45RO^{+}HLADR^{+}CCR4^{+} % of CD3^{+}CD4^{-}	T1D ↑	0.054	ns
CXCR3^{lo} (CD8 Tcm)	CD183^{lo} % of CD3^{+}CD4^{-} Tcm	T1D ↑	0.053	ns
MCHC	MCHC (g/dL)	T1D ↑	0.053	ns
CD38^{-}HLADR^{-} (CD8 Temra)	CD38^{-}HLADR^{-} % of CD8 Temra	T1D ↑	0.052	ns
CXCR5^{+} (CD8 Tem)	CD185^{+} % of CD8 Tem	T1D ↑	0.051	ns
PD1^{+} (CD8 Tem)	CD279^{+} % of CD8 Tem	T1D ↑	0.051	ns
CXCR3^{+} (Naive CD4)	CD183^{+} % of Naive CD4	T1D ↑	0.051	ns

HLADR^{+} (CD8CD45RO^{+}CXCR3^{-}CCR6^{-})	HLA^{+} % of CD4^{-}CD45RO^{+}CD183^{-}CD196^{-}	T1D ↑	0.05	ns
CXCR5^{+} (CD4 Temra)	CD185^{+} % of CD4 Temra	T1D ↑	0.049	ns
Memory Th2 (CD4)	CD45RO^{+}CD183^{-}CD196^{-} % of CD3^{+}CD4^{+}	T1D ↑	0.048	ns
PD1 index (Temra CD8)	Temra CD8 CD279mfi Rel. Diff.	T1D ↑	0.048	ns
Basophils (CBC)	BA (%)	T1D ↑	0.047	ns
CD4 (T cells)	CD4 % of T cells	T1D ↑	0.043	ns
HLADR^{+} (Memory Th17)	HLADR^{+} % of CD4^{+}CD45RO^{+}CD183^{-}CD196^{+}	T1D ↑	0.043	ns
Memory Th1/17 (CD4)	CD45RO^{+}CD183^{+}CD196^{+} % of CD3^{+}CD4^{+}	T1D ↑	0.042	ns
Activated Memory (Tconv)	CD45RO^{+}HLADR^{+}CCR4^{+} % of Tconv	T1D ↑	0.041	ns
CXCR3^{hi} (CD8 Temra)	CD183^{hi} % of CD3^{+}CD4^{-} Temra	T1D ↑	0.041	ns
Tfh (CD4^{+}CD45RA^{-}CXCR5^{+})	Tfh % of CD4^{+}CD45RA^{-}CD185^{+}	T1D ↑	0.041	ns
HLADR^{+}CD38^{+} (Memory Th1/17)	HLADR^{+}CD38^{+} % of CD4^{+}CD45RO^{+}CD183^{+}CD196^{+}	T1D ↑	0.036	ns
CXCR3^{lo} (CD8 Tem)	CD183^{lo} % of CD3^{+}CD4^{-} Tem	T1D ↑	0.034	ns
T cells (Lymphocytes)	T cells % of Lymphocytes	T1D ↑	0.034	ns
Non-Class-switched Memory (B cells)	Non-Class-switched Memory % of B cells	T1D ↑	0.034	ns
Tcm (CD8)	Tcm % of CD8	T1D ↑	0.032	ns
Memory Th17 (CD4)	CD45RO^{+}CD183^{-}CD196^{+} % of CD3^{+}CD4^{+}	T1D ↑	0.031	ns
Absolute neutrophil count	NE# (10^{3}/μL)	T1D ↑	0.031	ns
Memory Th1 (CD4)	CD45RO^{+}CD183^{+}CD196^{-} % of CD3^{+}CD4^{+}	T1D ↑	0.03	ns
Classical (Monocytes)	Classical % of Monocytes	T1D ↑	0.029	ns
HLADR^{+} (Memory Th1/17)	HLADR^{+} % of CD4^{+}CD45RO^{+}CD183^{+}CD196^{+}	T1D ↑	0.027	ns
CD38^{-}HLADR^{+} (CD4 Temra)	CD38^{-}HLADR^{+} % of CD4 Temra	T1D ↑	0.027	ns
HLADR^{+}CD38^{+} (CD8CD45RO^{+}CXCR3^{-}CCR6^{-})	HLA^{+}CD38^{+} % of CD4^{-}CD45RO^{+}CD183^{-}CD196^{-}	T1D ↑	0.026	ns
Monocytes (MNCs)	Monocytes % of MNCs	T1D ↑	0.024	ns
CD38^{+}HLADR^{+} (Naive CD8)	CD38^{+}HLADR^{+} % of Naive CD8	T1D ↑	0.024	ns
CD38^{-}HLADR^{-} (CD4 Tem)	CD38^{-}HLADR^{-} % of CD4 Tem	T1D ↑	0.023	ns
Tfh (CD4)	Tfh % of CD4	T1D ↑	0.023	ns
CD8 (T cells)	CD8 % of T cells	T1D ↑	0.022	ns
CXCR5^{+} (CD4 Tem)	CD185^{+} % of CD4 Tem	T1D ↑	0.02	ns
PD1 index (Tem CD8)	Tem CD8 CD279mfi Rel. Diff.	T1D ↑	0.019	ns
Absolute basophil count	BA# (10^{3}/μL)	T1D ↑	0.017	ns
CD38^{+}HLADR^{+} (CD8)	CD38^{+}HLADR^{+} % of CD8	T1D ↑	0.017	ns
Precursor Tfh (CD4^{+}CD45RA^{-}CXCR5^{+})	Precursor Tfh % of CD4^{+}CD45RA^{-}CD185^{+}	T1D ↑	0.017	ns
Tem (CD4)	Tem % of CD4	T1D ↑	0.017	ns
CD38^{-}HLADR^{-} (CD4)	CD38^{-}HLADR^{-} % of CD4	T1D ↑	0.017	ns
CD25mfi (CD45RO^{-} Tconv)	CD25mfi of CD45RO^{-} Tconv	T1D ↑	0.017	ns
CD38^{-}HLADR^{+} (CD8 Tcm)	CD38^{-}HLADR^{+} % of CD8 Tcm	T1D ↑	0.016	ns

$HLADR^+$ ($CD8CD45RO^+CXCR3^+CCR6^-$)	HLA^+ % of $CD4^-CD45RO^+CD183^+CD196^-$	T1D ↑	0.016	ns
$CD38^+HLADR^-$ (CD4 Tcm)	$CD38^+HLADR^-$ % of CD4 Tcm	T1D ↑	0.015	ns
$PD1^+$ (CD4 Tcm)	$CD279^+$ % of CD4 Tcm	T1D ↑	0.015	ns
DC (MNCs)	DC % of MNCs	T1D ↑	0.015	ns
$CD38^+HLADR^+$ (CD4 Temra)	$CD38^+HLADR^+$ % of CD4 Temra	T1D ↑	0.014	ns
$CD38^+HLADR^+$ (CD8 Tem)	$CD38^+HLADR^+$ % of CD8 Tem	T1D ↑	0.014	ns
$HLADR^+CD38^+$ (Memory Th2)	$HLADR^+CD38^+$ % of $CD4^+CD45RO^+CD183^-CD196^-$	T1D ↑	0.013	ns
$CD38^-HLADR^-$ (CD4 Temra)	$CD38^-HLADR^-$ % of CD4 Temra	T1D ↑	0.012	ns
$HLADR^+CD38^+$ ($CD8CD45RO^+CXCR3^+CCR6^-$)	HLA^+CD38^+ % of $CD4^-CD45RO^+CD183^+CD196^-$	T1D ↑	0.011	ns
Precursor Tfh (CD4)	Precursor Tfh % of CD4	T1D ↑	0.009	ns
$CD38^-HLADR^+$ (CD8 Tem)	$CD38^-HLADR^+$ % of CD8 Tem	T1D ↑	0.009	ns
$CXCR3^+$ (CD4 Tem)	$CD183^+$ % of CD4 Tem	T1D ↑	0.008	ns
$CD38^+HLADR^+$ (CD4 Tem)	$CD38^+HLADR^+$ % of CD4 Tem	T1D ↑	0.007	ns
$CD38^+HLADR^+$ (Naive CD4)	$CD38^+HLADR^+$ % of Naive CD4	T1D ↑	0.006	ns
B cells (Lymphocytes)	B cells % of Lymphocytes	T1D ↑	0.005	ns
WBC count	WBC (10^3/μL)	T1D ↑	0.005	ns
$CXCR3^+$ (CD4 Temra)	$CD183^+$ % of CD4 Temra	T1D ↑	0.005	ns
$CD38^-HLADR^+$ (CD4 Tcm)	$CD38^-HLADR^+$ % of CD4 Tcm	T1D ↑	0.005	ns
CD25mfi (Naïve CD8)	CD25mfi of $CD45RO^-CD3^+CD4^-$	T1D ↑	0.005	ns
$CXCR3^+$ (CD8 Tcm)	$CD183^+$ % of CD8 Tcm	T1D ↑	0.004	ns
$CD38^-HLADR^+$ (Naive CD8)	$CD38^-HLADR^+$ % of Naive CD8	T1D ↑	0.004	ns
$HLADR^+CD38^+$ ($CD8CD45RO^+CXCR3^{lo}CCR6^+$)	HLA^+CD38^+ % of $CD4^-CD45RO^+CD183^{lo}CD196^+$	T1D ↑	0.003	ns
$CD38^-HLADR^+$ (CD4)	$CD38^-HLADR^+$ % of CD4	T1D ↑	0.003	ns
CD25mfi (Memory CD8)	CD25mfi of $CD45RO^+CD3^+CD4^-$	T1D ↑	0.003	ns
$CD38^+HLADR^+$ (CD4)	$CD38^+HLADR^+$ % of CD4	T1D ↑	0.001	ns
$CD38^-HLADR^+$ (CD4 Tem)	$CD38^-HLADR^+$ % of CD4 Tem	T1D ↑	0.001	ns
$PD1^+$ (CD4 Temra)	$CD279^+$ % of CD4 Temra	T1D ↑	0	ns
$CXCR3^{lo}CCR6^+$ (CD8)	$CD183^{lo}CD196^+$ % of $CD3^+CD4^-$	T1D ↑	0	ns
$CD38^+HLADR^+$ (CD8 Tcm)	$CD38^+HLADR^+$ % of CD8 Tcm	T1D ↑	0	ns
$CD38^-HLADR^-$ (CD8 Tem)	$CD38^-HLADR^-$ % of CD8 Tem	T1D ↓	0.001	ns
$HLADR^+CD38^+$ (Memory Th1)	$HLADR^+CD38^+$ % of $CD4^+CD45RO^+CD183^+CD196^-$	T1D ↓	0.001	ns
DP (T cells)	DP % of T cells	T1D ↓	0.002	ns
$CD38^+HLADR^-$ (CD8 Tcm)	$CD38^+HLADR^-$ % of CD8 Tcm	T1D ↓	0.002	ns
Treg (CD4)	Treg % of $CD4^+$	T1D ↓	0.003	ns
Conventional (DCs)	$CD11c^-CD123^-$ % of DC	T1D ↓	0.006	ns
$CXCR5^+$ (Naive CD8)	$CD185^+$ % of Naive CD8	T1D ↓	0.006	ns
$CD16^+$ ($CD56^{bright}$ NK)	$CD16^+$ % of $CD56^{bright}$ NK	T1D ↓	0.007	ns

PD1mfi (Temra CD8)	Temra CD8 CD279mfi	T1D ↓	0.008	ns
Absolute monocyte count	MO# (10^3/µL)	T1D ↓	0.009	ns
$CD38^+HLADR^+$ (CD8 Temra)	$CD38^+HLADR^+$ % of CD8 Temra	T1D ↓	0.011	ns
Memory Treg CD25 index	$CD45RO^+$ Treg CD25mfi Rel. Diff.	T1D ↓	0.013	ns
$CD38^+HLADR^+$ (CD4 Tcm)	$CD38^+HLADR^+$ % of CD4 Tcm	T1D ↓	0.014	ns
$HLADR^+$ ($CD8CD45RO^+CXCR3^{lo}CCR6^+$)	HLA^+ % of $CD4^-CD45RO^+CD183^{lo}CD196^+$	T1D ↓	0.014	ns
$CXCR5^+$ (CD4 Tcm)	$CD185^+$ % of CD4 Tcm	T1D ↓	0.015	ns
$CXCR3^-$ (CD8 Tem)	$CD183^-$ % of $CD3^+CD4^-$ Tem	T1D ↓	0.015	ns
$CXCR5^+$ (CD8 Tcm)	$CD185^+$ % of CD8 Tcm	T1D ↓	0.016	ns
CD25mfi ($CD45RO^+$ Tconv	CD25mfi of $CD45RO^+$ Tconv	T1D ↓	0.016	ns
$CD38^+HLADR^-$ (CD4)	$CD38^+HLADR^-$ % of CD4	T1D ↓	0.017	ns
$CXCR3^+$ (CD8)	$CD183^+CD196^-$ % of $CD3^+CD4^-$	T1D ↓	0.018	ns
$PD1^+$ (CD8 Tcm)	$CD279^+$ % of CD8 Tcm	T1D ↓	0.018	ns
Memory CD8 CD25 index	$CD45RO^+CD3^+CD4^-$ CD25mfi Rel. Diff.	T1D ↓	0.019	ns
$CD38^-HLADR^-$ (CD8 Tcm)	$CD38^-HLADR^-$ % of CD8 Tcm	T1D ↓	0.02	ns
$HLADR^+$ (Memory Th2)	$HLADR^+$ % of $CD4^+CD45RO^+CD183^-CD196^-$	T1D ↓	0.022	ns
$HLADR^+$ (Memory Th1)	$HLADR^+$ % of $CD4^+CD45RO^+CD183^+CD196^-$	T1D ↓	0.022	ns
$CD38^-HLADR^-$ (CD4 Tcm)	$CD38^-HLADR^-$ % of CD4 Tcm	T1D ↓	0.024	ns
Memory Tconv CD25 index	$CD45RO^+$ Tconv CD25mfi Rel. Diff.	T1D ↓	0.024	ns
$CD38^-HLADR^+$ (CD8)	$CD38^-HLADR^+$ % of CD8	T1D ↓	0.026	ns
$CXCR3^-$ (CD8 Tcm)	$CD183^-$ % of $CD3^+CD4^-$ Tcm	T1D ↓	0.027	ns
$CXCR3^{hi}$ (CD8 Tem)	$CD183^{hi}$ % of $CD3^+CD4^-$ Tem	T1D ↓	0.027	ns
Memory Tfh (CD4)	Memory Tfh % of CD4	T1D ↓	0.028	ns
Non-Classical (Monocytes)	Non-Classical % of Monocytes	T1D ↓	0.029	ns
$CXCR3^{hi}$ (CD8 Tcm)	$CD183^{hi}$ % of $CD3^+CD4^-$ Tcm	T1D ↓	0.029	ns
Absolute eosinophil count	EO# (10^3/µL)	T1D ↓	0.032	ns
Memory Tfh ($CD4^+CD45RA^-CXCR5^+$)	Memory Tfh % of $CD4^+CD45RA^-CD185^+$	T1D ↓	0.032	ns
Eosinophils (CBC)	EO (%)	T1D ↓	0.032	ns
PD1mfi (Tem CD8)	Tem CD8 CD279mfi	T1D ↓	0.032	ns
$PD1^+$ (CD4 Tem)	$CD279^+$ % of CD4 Tem	T1D ↓	0.034	ns
Temra (CD4)	Temra % of CD4	T1D ↓	0.036	ns
Monocytes (CBC)	MO (%)	T1D ↓	0.036	ns
PD1 index (Temra CD4)	Temra CD4 CD279mfi Rel. Diff.	T1D ↓	0.039	ns
MPV	MPV [mean platelet volume] (fL)	T1D ↓	0.039	ns
Class-switched Memory (B cells)	Class-switched Memory % of B cells	T1D ↓	0.04	ns
$CXCR3^+$ (CD8 Tem)	$CD183^+$ % of CD8 Tem	T1D ↓	0.041	ns
$CXCR3^{hi}$ (CD8 Naïve)	$CD183^{hi}$ % of $CD3^+CD4^-$ Naive	T1D ↓	0.041	ns

$CD38^+HLADR^-$ (CD8 Tem)	$CD38^+HLADR^-$ % of CD8 Tem	T1D ↓	0.042	ns
$CD38^-HLADR^+$ (Naive CD4)	$CD38^-HLADR^+$ % of Naive CD4	T1D ↓	0.043	ns
Naive (CD4)	Naive % of CD4	T1D ↓	0.047	ns
$CXCR3^-CCR6^-$ (CD8)	$CD183^-CD196^-$ % of $CD3^+CD4^-$	T1D ↓	0.048	ns
$CD38^+HLADR^-$ (CD4 Tem)	$CD38^+HLADR^-$ % of CD4 Tem	T1D ↓	0.049	ns
PD1 index (Tcm CD8)	Tcm CD8 CD279mfi Rel. Diff.	T1D ↓	0.05	ns
$CD38^+HLADR^-$ (CD4 Temra)	$CD38^+HLADR^-$ % of CD4 Temra	T1D ↓	0.052	ns
PD1mfi (Naive CD4)	Naive CD4 CD279mfi	T1D ↓	0.053	ns
Temra (CD8)	Temra % of CD8	T1D ↓	0.055	ns
Plasmacytoid (DCs)	Plasmacytoid % DCs	T1D ↓	0.055	ns
PD1mfi (Naive CD8)	Naive CD8 CD279mfi	T1D ↓	0.056	ns
CD25mfi (Memory Treg)	CD25mfi of $CD45RO^+$ Treg	T1D ↓	0.057	ns
CD25mfi (Naïve Treg)	CD25mfi of $CD45RO^-$ Treg	T1D ↓	0.059	ns
Absolute lymphocyte count	LY# (10^3/µL)	T1D ↓	0.06	ns
Plasmablast (B cells)	Plasmablast/Plasma Cell % of B cells	T1D ↓	0.065	ns

Table S5. Logistic regression of Genotype-Tissue Expression project (GTeX) *PDCD1* expression quantitative trait loci (eQTL) in CTR versus T1D subjects. Association between single nucleotide polymorphisms (SNP) and clinical status tested with sex and population stratification as covariates. A1 = effect allele, A2 = non-effect allele, OR = odds ratio, STAT = coefficient t-statistic, P = p-value. (See also Figure S15)

SNP	A1	A2	OR	STAT	P
rs6422701	C	T	0.627	-2.431	0.015
rs35905226	T	C	1.616	2.344	0.019
rs34623950	G	A	1.501	2.019	0.044
rs28435574	A	G	1.454	1.911	0.056
rs6734491	T	C	1.443	1.865	0.062
rs35380510	T	C	1.401	1.637	0.102
rs11683104	C	T	1.345	1.486	0.137
rs67309595	A	G	1.331	1.458	0.145
rs4596012	C	T	0.754	-1.424	0.155
rs35301767	A	G	0.750	-1.322	0.186
rs73108258	A	G	0.649	-1.112	0.266
rs66492541	A	G	1.169	0.787	0.432
rs6422702	A	G	0.846	-0.780	0.436
rs78280775	C	T	0.890	-0.573	0.567
rs10755057	T	G	0.928	-0.364	0.716
rs35350837	A	G	0.937	-0.322	0.748
rs1106416	T	C	1.125	0.306	0.760
rs79326616	T	C	0.917	-0.300	0.764
rs74898709	A	G	0.923	-0.295	0.768
rs78415814	T	C	0.918	-0.287	0.774
rs114496196	C	A	0.904	-0.244	0.808
rs74603454	A	C	0.948	-0.196	0.845
rs1106639	A	G	1.026	0.119	0.905
rs10933569	A	G	1.003	0.014	0.989

Table S6. QTL analysis. Linear regression analysis of associations between 277 T1D genetic risk loci and 172 GAMLSS-corrected flow cytometric parameters, considering T1D status, sex, and population admixture as covariates. Beta coefficients are shown in the context of the T1D risk allele. False discovery rate (FDR) for total genotype and phenotype combinations determined by Benjamini-Hochberg method. Data are available in a supplementary Excel file. (See also Figure 7).

Table S7. Reagents and resources used in the study.

REAGENT or RESOURCE	SOURCE	IDENTIFIER
**Antibodies**		
Pacific Blue anti-CD3	BioLegend	Cat#300417; RRID:AB_493094
Pacific Blue anti-CD14	BioLegend	Cat#301828; RRID:AB_2275670
BV421 anti-CD197	Biolegend	Cat#353208; RRID:AB_10915137
FITC anti-CD4	Biolegend	Cat#300506; RRID:AB_314074
AF488 anti-HLA-DR	BioLegend	Cat#307620; RRID:AB_493175
AF488 anti-IgD	BioLegend	Cat#348215; RRID:AB_11150397
PerCP/Cy5.5 anti-CD4	BioLegend	Cat#300530; RRID:AB_893322
PerCP/Cy5.5 anti-CD19	BioLegend	Cat#302230; RRID:AB_2073119
PerCP/Cy5.5 anti-CD45RA	Biolegend	Cat#304122; RRID:AB_893357
PerCP/Cy5.5 anti-CD123	Biolegend	Cat#306016; RRID:AB_2264693
PE anti-CD24	Biolegend	Cat#311106; RRID:AB_314855
PE anti-CD56	Biolegend	Cat#318306; RRID:AB_604101
PE anti-CD127	BioLegend	Cat#351304; RRID:AB_10720185
PE anti-CD183	BioLegend	Cat#353706; RRID:AB_10962912
PE anti-CD185	BioLegend	Cat#356904; RRID:AB_2561813
PE anti-CD197	BioLegend	Cat#353204; RRID:AB_10913813
PE/Cy7 anti-CD11c	BioLegend	Cat#337216; RRID:AB_2129790
PE/Cy7 anti-CD27	BioLegend	Cat#302838; RRID:AB_2561919
PE/Cy7 anti-CD45RA	BioLegend	Cat#304126; RRID:AB_10708879
PE/Cy7 anti-CD183	BioLegend	Cat#353720; RRID:AB_11219383
PE/Cy7 anti-CD194	BioLegend	Cat#359410; RRID:AB_2562431
PE/Cy7 anti-CD196	BioLegend	Cat#353418; RRID:AB_10916518
APC anti-CD16	BioLegend	Cat#302012; RRID:AB_314212
APC anti-CD25	BioLegend	Cat#302610; RRID:AB_314280
APC anti-CD38	BioLegend	Cat#356606; RRID:AB_2561902
APC anti-CD279	eBioscience	Cat#17-2799-42; RRID:AB_11063701
APC/H7 anti-CD3	BD Biosciences	Cat#560176; RRID:AB_1645475
APC/H7 anti-CD8	BD Biosciences	Cat#560179; RRID:AB_1645481
APC/H7 anti-CD19	BD Biosciences	Cat#560727; RRID:AB_1727437
APC/H7 anti-CD20	BD Biosciences	Cat#560853; RRID:AB_10561681
APC/H7 anti-CD45RO	BD Biosciences	Cat#561137; RRID:AB_10562194
Human TruStain FcX™	BioLegend	Cat#422302; RRID:AB_2818986
True-Stain Monocyte Blocker	BioLegend	Cat#426103
**Critical Commercial Assays**		
eBioscience 1-step Fix/Lyse Solution 10X Concentrate	ThermoFisher	Cat#00-5333-54
**Software and Algorithms**		
FlowJo v9 and v10	BD Life Sciences	https://www.flowjo.com

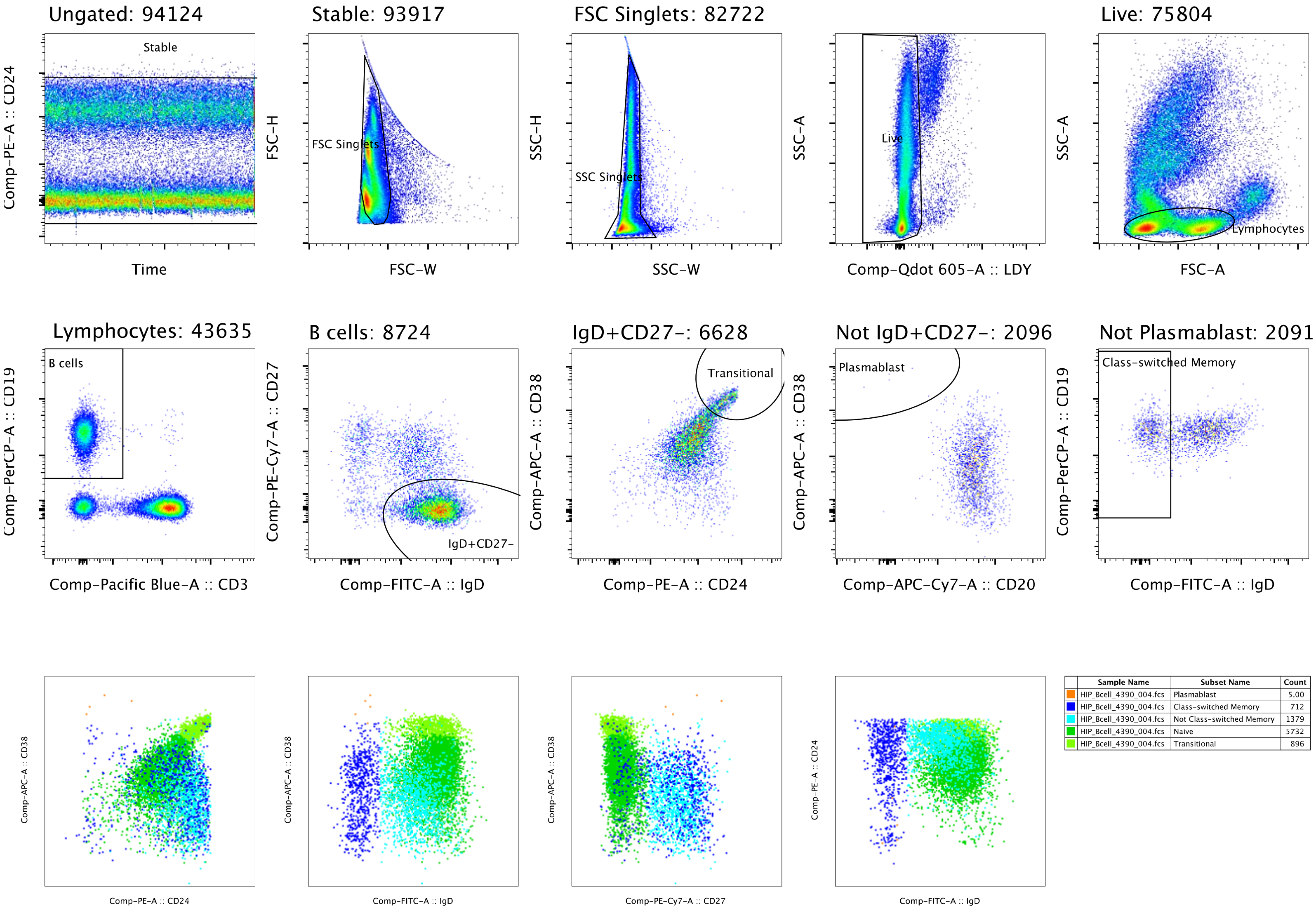

	Sample Name	Subset Name	Count
	HIP_Bcell_4390_004.fcs	Plasmablast	5.00
	HIP_Bcell_4390_004.fcs	Class-switched Memory	712
	HIP_Bcell_4390_004.fcs	Not Class-switched Memory	1379
	HIP_Bcell_4390_004.fcs	Naive	5732
	HIP_Bcell_4390_004.fcs	Transitional	896

Figure S1. B cell panel gating. Gating based on time, forward scatter (FSC), and side scatter (SSC) was performed to select stably

acquired singlets. The Qdot605 channel was used to remove autofluorescent granulocytes, then lymphocytes were gated based on FSC and SSC parameters. $CD19^{+}CD3^{-}$ B cells were subset into $IgD^{+}CD27^{-}$ cells, comprised of $CD24^{lo/-}CD38^{lo/-}$ naïve (dark green) and $CD24^{hi}CD38^{hi}$ transitional (light green) B cells. B cells that were not in the $IgD^{+}CD27^{-}$ gate were subset into $CD20^{-}CD38^{hi}$ plasmablasts (orange). Non-plasmablasts were designated as IgD^{+} non-class-switched (light blue) or IgD^{-} class-switched memory (dark blue) B cells.

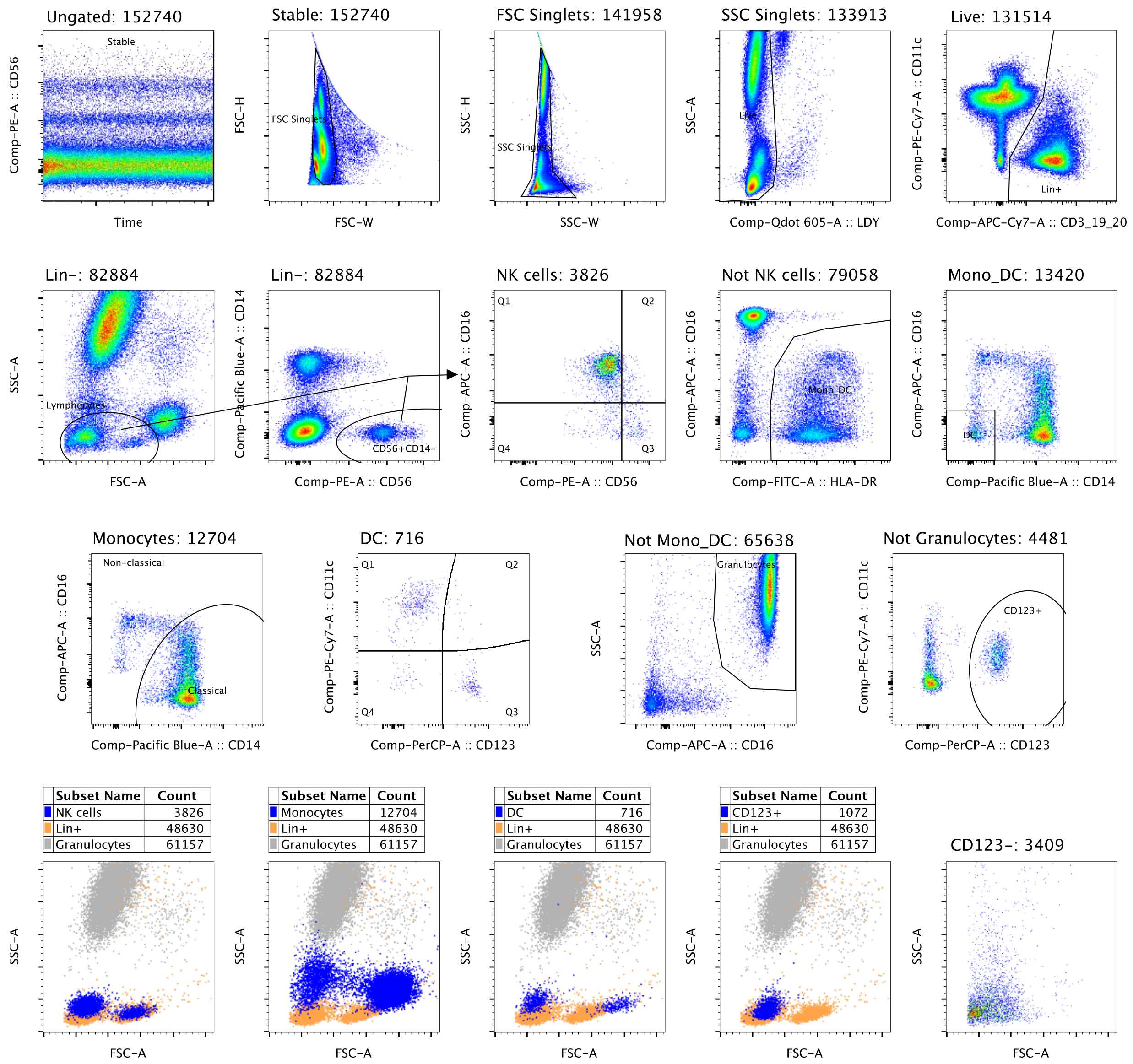

Figure S2. Innate immune cell panel gating. Gating based on time, forward scatter (FSC), and side scatter (SSC) was performed to select stably acquired singlets. The Qdot605/LDY channel was used to remove autofluorescent granulocytes, then lineage negative (Lin^{-}) CD3^{-}CD19^{-}CD20^{-} cells were selected. Lymphocytes were gated based on FSC and SSC parameters, followed by gating on CD56^{+}CD14^{-} natural killer (NK) cells (blue, bottom left). NK cells were quadrant gated into CD56^{bright}, CD56^{dim}, and CD16^{+} of CD56^{bright} subsets. HLA-DR^{+} non-NK cells were subset into CD14^{-}CD16^{-} dendritic cells (DCs, blue, bottom middle), with non-DC as monocytes (blue,

bottom second to left). Monocytes were grouped into $CD14^{+}CD16^{-}$ classical and $CD14^{dim}CD16^{+}$ non-classical subsets, while DC were grouped into $CD11c^{+}CD123^{-}$ myeloid, $CD11c^{-}CD123^{+}$ plasmacytoid, and $CD11c^{-}CD123^{-}$ conventional subsets. Non-monocyte/DC were gated for $SSC^{hi}CD16^{hi}$ granulocytes and non-granulocytes for other undefined $CD123^{+}$ mononuclear cells (MNCs) (blue, bottom second to right). Size and granularity of previously defined major innate subsets compared to lineage positive (Lin^{+}) cells (orange).

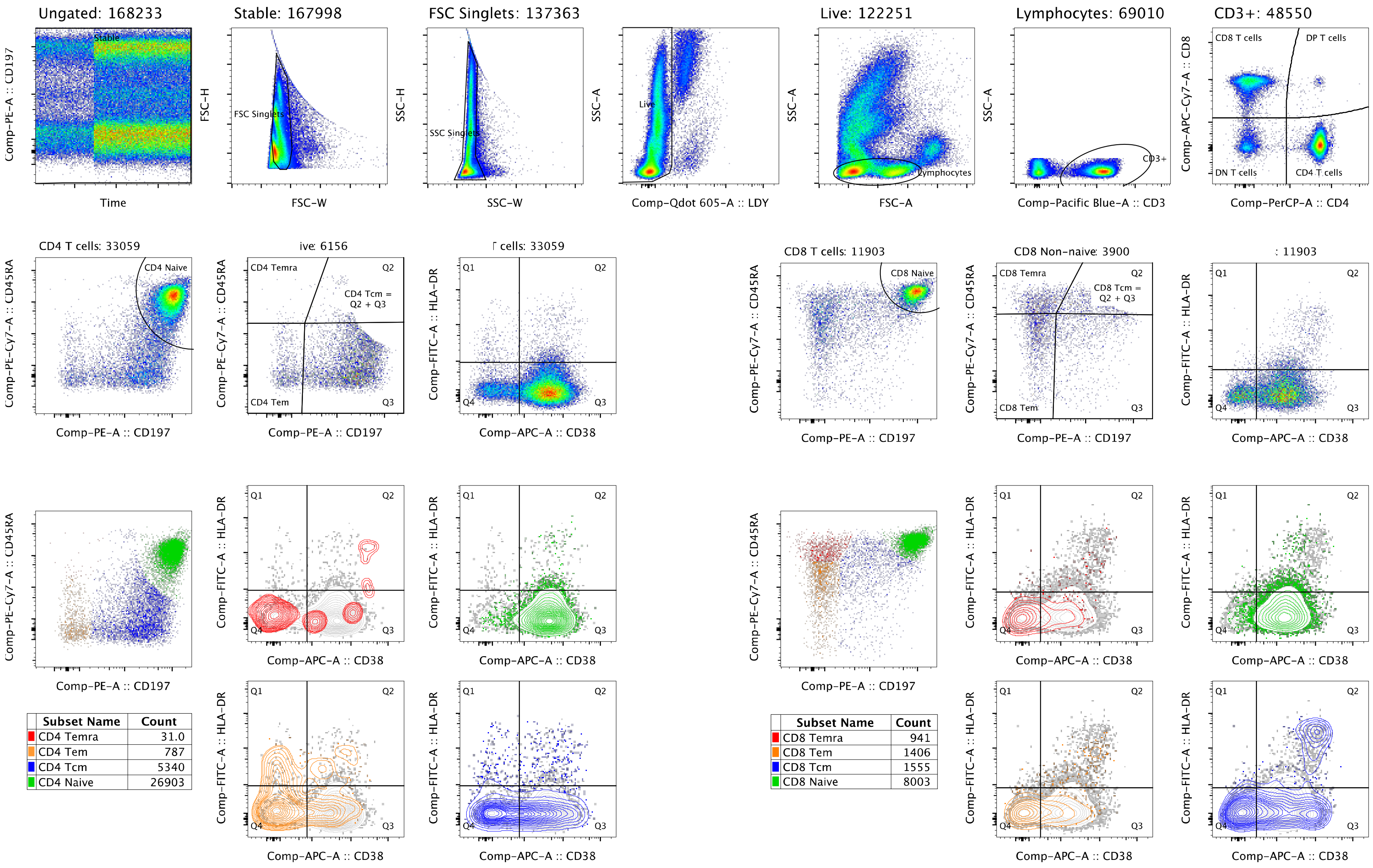

Subset Name	Count
CD4 Temra	31.0
CD4 Tem	787
CD4 Tcm	5340
CD4 Naive	26903

Subset Name	Count
CD8 Temra	941
CD8 Tem	1406
CD8 Tcm	1555
CD8 Naive	8003

Figure S3. T cell panel gating. Gating based on time, forward scatter (FSC), and side scatter (SSC) was performed to select stably acquired singlets. The Qdot605/LDY channel was used to remove autofluorescent granulocytes, then lymphocytes were gated based on

FSC and SSC parameters. $CD3^+$ T cells were grouped into $CD4^+$, $CD8^+$, double negative (DN, $CD4^-CD8^-$), and double positive (DP, $CD4^+CD8^+$) T cell subsets. Gating was performed within $CD4^+$ (left) and $CD8^+$ (right) T cell populations to select $CD45RA^+CD197^+$ naïve (green), $CD45RA^-CD197^+$ central memory (Tcm, blue), $CD45RA^-CD197^-$ effector memory (Tem, orange), and $CD45RA^+CD197^-$ terminally differentiated effector memory (Temra, red) T cells. $CD38^-$HLA-DR^-, $CD38^+$HLA-DR^-, $CD38^-$HLA-DR^+, and $CD38^+$HLA-DR^+ subsets were gated within total as well as naïve, Tcm, Tem, and Temra CD4 or CD8 T cells.

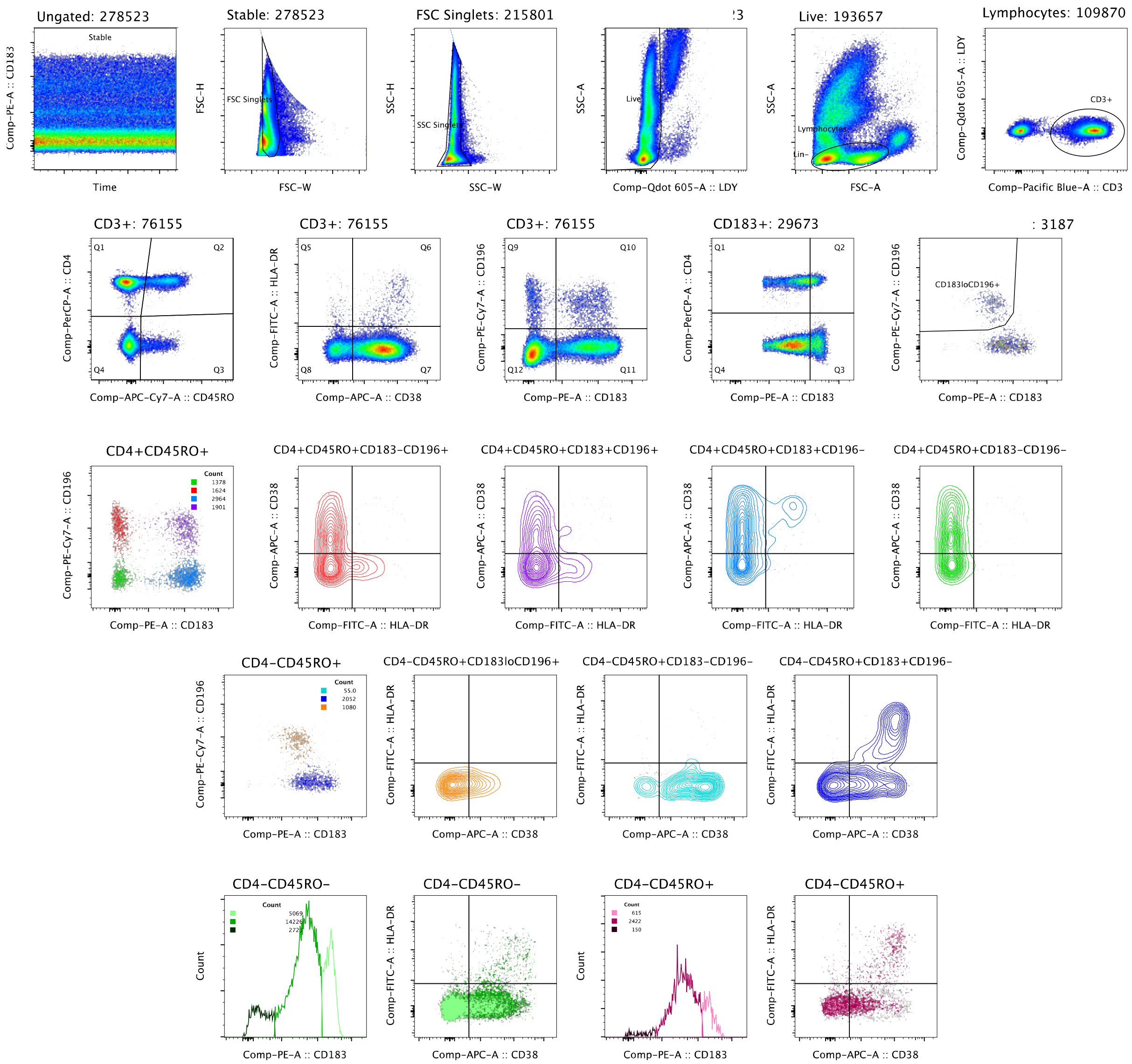

Figure S4. Effector T cell (Teff) panel gating. Gating based on time, forward scatter (FSC), and side scatter (SSC) was performed to select stably acquired singlets. The Qdot605/LDY channel was used to remove autofluorescent granulocytes, then lymphocytes were gated based on FSC and SSC parameters. $CD3^+$ T cells were grouped into $CD4^+CD45RO^+$ memory T cells, comprising $CD183^-CD196^+$ Th17 (red), $CD183^+CD196^+$ Th1/17 (purple), $CD183^+CD196^-$ Th1 (blue), and $CD183^-CD196^-$ Th2 (green) subsets. $CD3^+CD4^-CD45RO^+$ memory CD8 T cells were subset into $CXCR3^{lo}CCR6^+$ (orange), $CXCR3^-CCR6^-$ (light blue), and $CXCR3^+CCR6^-$ (dark blue)

populations. The previous seven cell subsets were further divided into HLA-DR^+ and HLA-DR^+CD38^+ portions. $CD3^+CD4^-CD45RO^-$ naïve CD8 T cells were gated to $CXCR3^-$ (dark green), $CXCR3^{lo}$ (green), $CXCR3^{hi}$ (light green) subsets. $CD3^+CD4^-CD45RO^+$ memory CD8 T cell populations shown as $CXCR3^-$ (dark purple), $CXCR3^{lo}$ (purple), and $CXCR3^{hi}$ (pink).

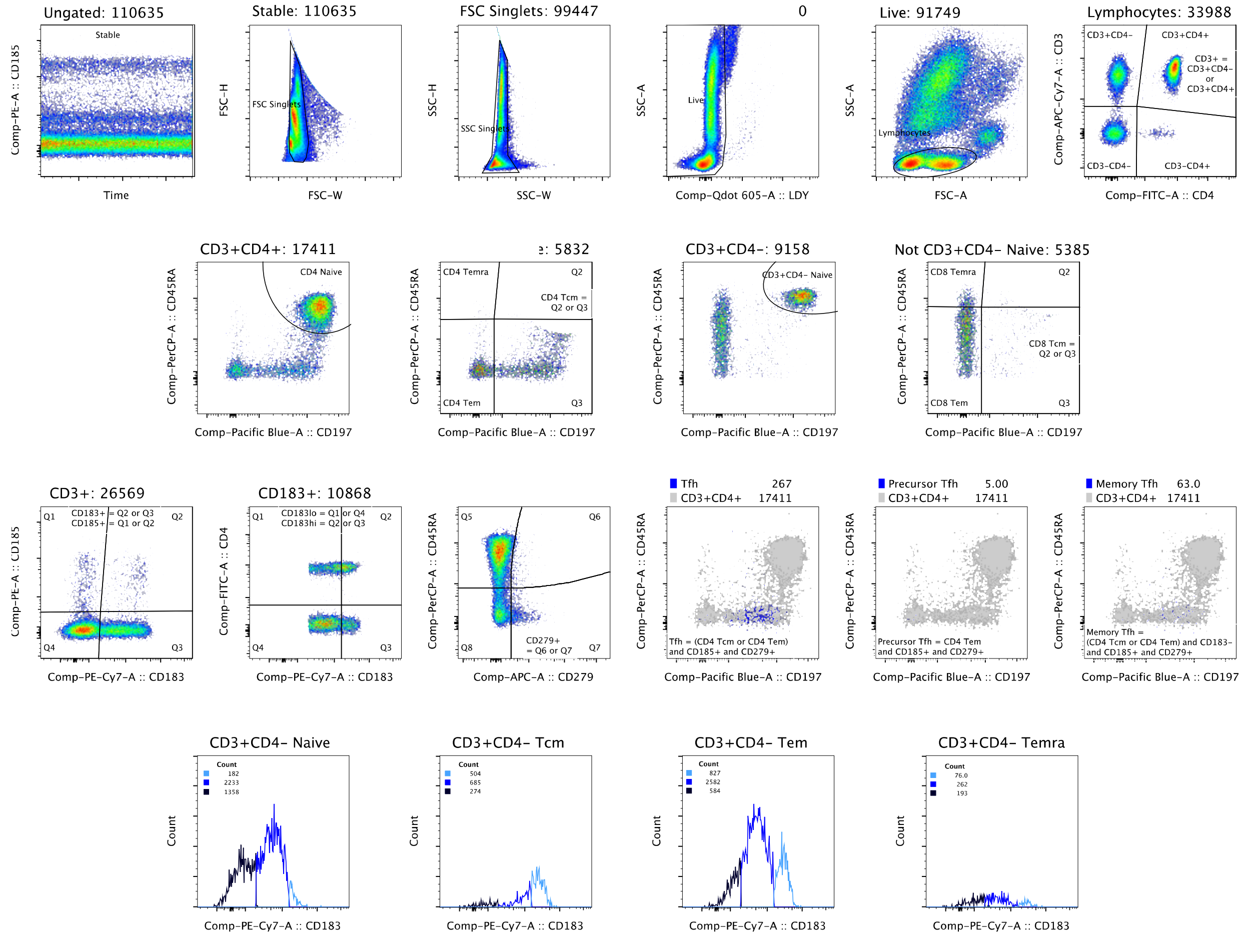

Figure S5. T follicular helper cell (Tfh) panel gating. Gating based on time, forward scatter (FSC), and side scatter (SSC) was performed to select stably acquired singlets. The Qdot605/LDY channel was used to remove autofluorescent granulocytes, then lymphocytes were gated based on FSC and SSC parameters. CD3$^+$CD4$^+$ and CD3$^+$CD4$^-$ T cells were grouped into naïve, Tcm, Tem, and Temra based on CD45RA and CD197 expression (see Figure S3). PD-1/CD279$^+$, CXCR3/CD183$^+$, and CXCR5/CD185$^+$ populations were gated from the previously defined CD4 and CD8 T cell subsets. CD4 T cells were gated to CD45RA$^-$CXCR5$^+$PD-1$^+$ T follicular helper (Tfh). Tfh were subset into CCR7/CD197$^-$ precursor Tfh, CCR7/CD197$^+$CD183/CXCR3$^-$ memory Tfh, and CXCR3/CD183$^+$ Tfh populations. Additionally, CXCR3/CD183$^-$ (dark blue),

CXCR3/CD183^{lo} (blue), and CXCR3/CD183^{hi} (light blue) populations were determined based on histograms of marker expression as shown, equivalent to method in Fig.S4.

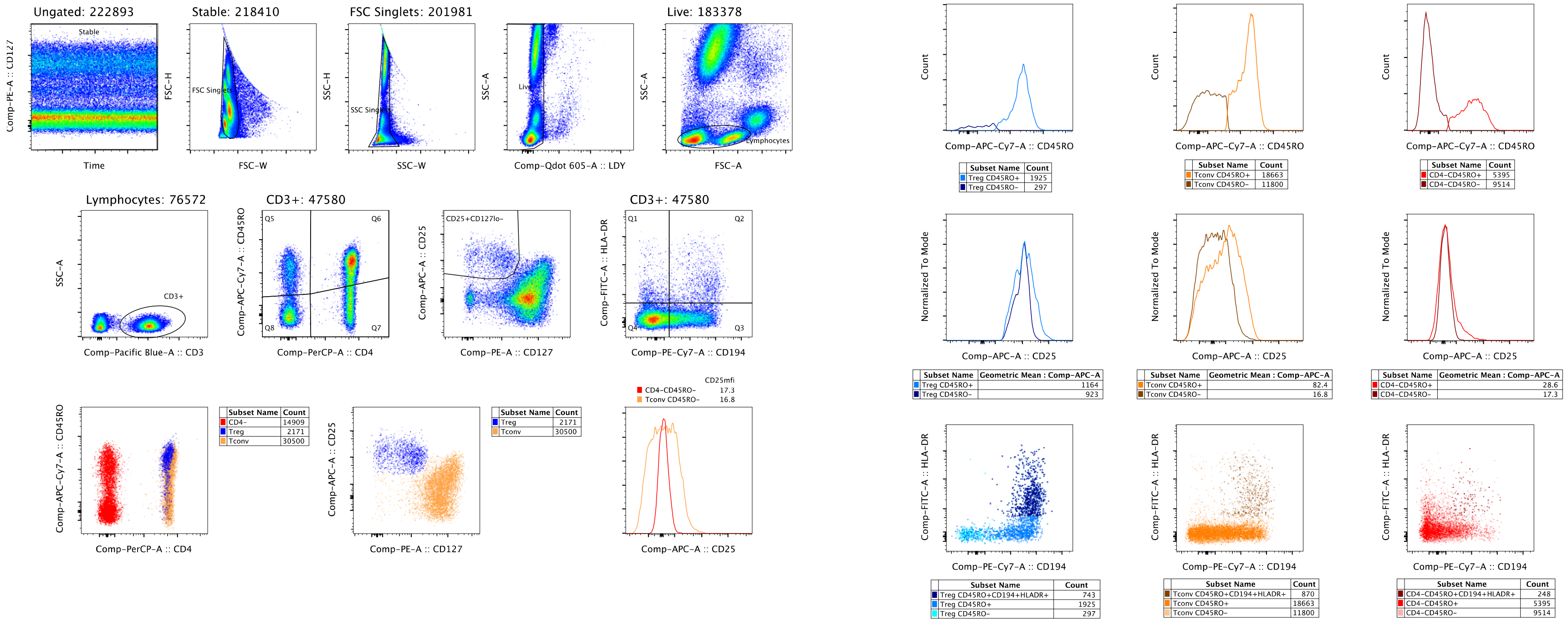

Figure S6. Regulatory T cell (Treg) panel gating. Gating based on time, forward scatter (FSC), and side scatter (SSC) was performed to select stably acquired singlets. The Qdot605/LDY channel was used to remove autofluorescent granulocytes, then lymphocytes were gated based on FSC and SSC parameters. Bottom left: $CD3^{+}$ T cells were grouped into $CD4^{+}CD25^{hi}CD127^{lo/-}$ Treg (blue), $CD4^{+}$ Tconv as non-Treg (orange), and $CD4^{-}$ to denote CD8 T cells (red). Bottom middle: histogram of CD25 expression on $CD3^{+}CD4^{-}CD45RO^{-}$ naïve CD8 (red, bottom middle) and $CD45RO^{-}$ naïve Tconv (orange) demonstrates compensation strategy. Right top/middle: CD45RO and CD25 expression on $CD45RO^{+}$ memory Treg (light blue), $CD45RO^{-}$ naïve Treg (blue), $CD45RO^{+}$ memory Tconv (orange),

$CD45RO^-$ naïve Tconv (brown), $CD3^+CD4^-CD45RO^+$ memory CD8 (red), and $CD3^+CD4^-CD45RO^-$ naïve CD8 (dark red) T cells. Right bottom: CCR4/CD194 and HLA-DR expression on $CD45RO^+CCR4^+HLA\text{-}DR^+$ activated memory Treg (dark blue), $CD45RO^+$ memory Treg (blue), $CD45RO^-$ naïve Treg (light blue), $CD45RO^+CCR4^+HLA\text{-}DR^+$ Tconv/memory Th2 (brown), $CD45RO^+$ memory Tconv (orange), $CD45RO^-$ naïve Tconv (light orange), $CD45RO^+CCR4^+HLA\text{-}DR^+$ CD8 (dark red), $CD4^-CD45RO^+$ memory CD8 (red), and $CD45RO^-$ naïve CD8 (pink) T cells.

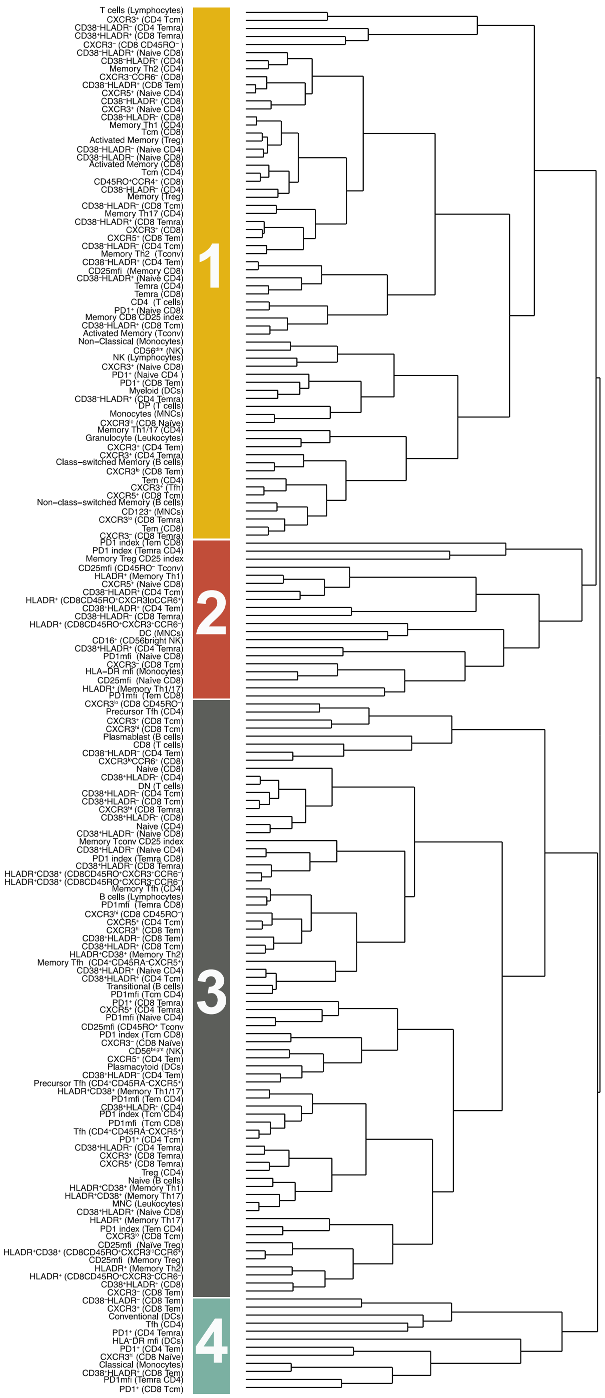

Figure S7. Dendrogram of phenotype age trajectories. Hierarchical clustering of the fitted phenotypic age trajectories resulted in a high-level splitting into four major groups. (See also Figure 2).

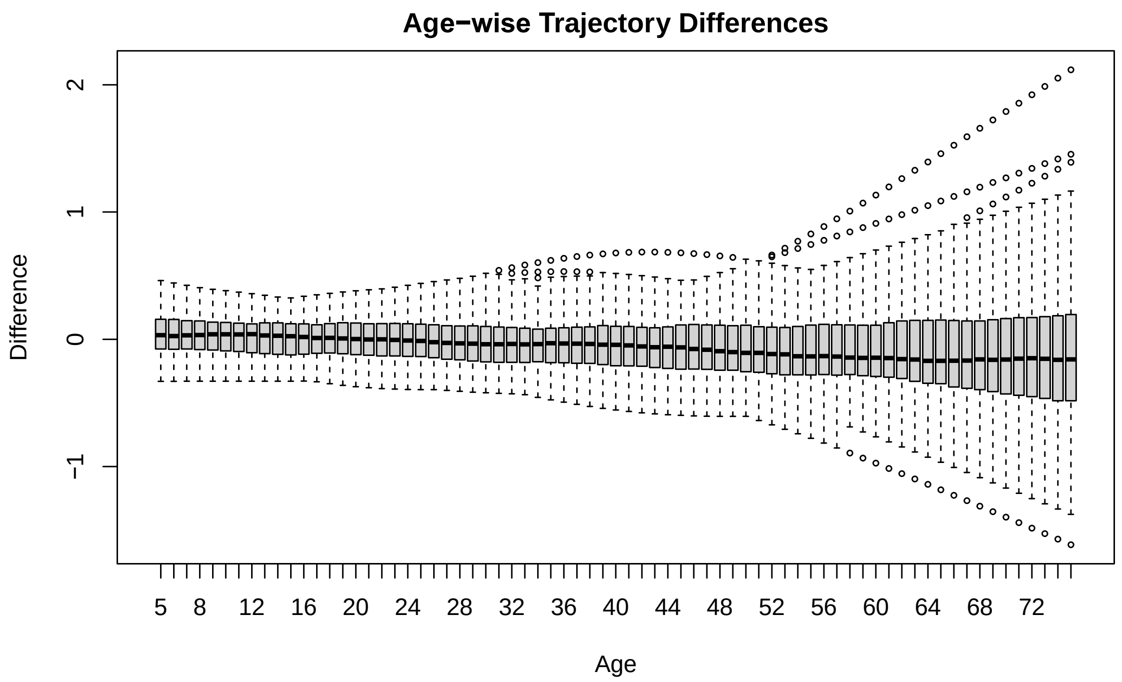

Figure S8. Age-wise trajectory differences. The differences in the estimated trajectory for T1D versus AAb- individuals for each phenotype are shown as boxplots for ages 5-75. (See also Figure 2).

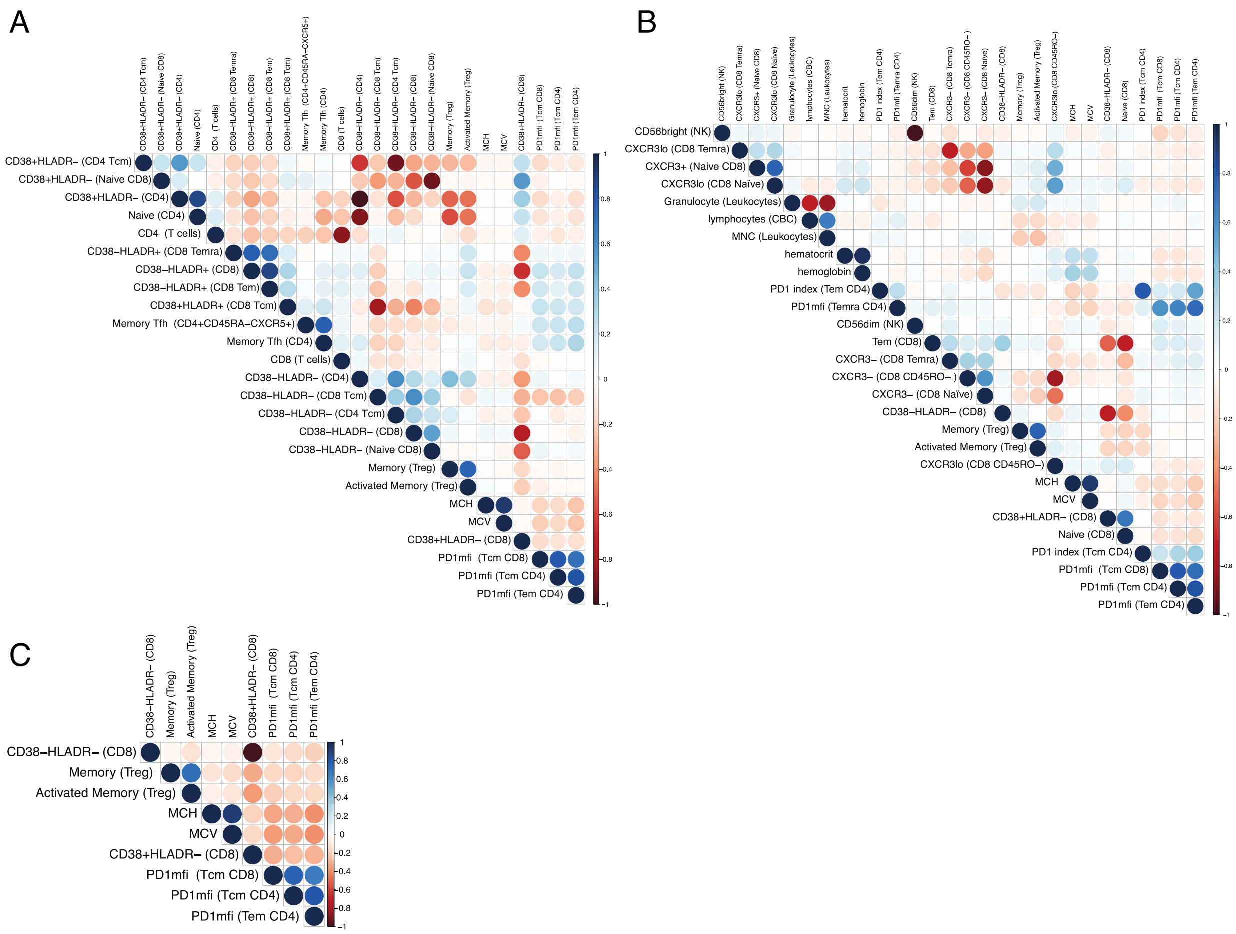

Figure S9. Correlations among significant age-corrected phenotypes. (**A**) Pairwise Spearman correlation of age-corrected phenotypes that are significantly associated with age and have an absolute correlation larger than 0.7 are shown. Positive correlations are displayed as various shades of blue and negative correlations are indicated by shades of red. (**B**) Similar to A for phenotypes significantly associated with T1D. (**C**) Similar to A for phenotypes significant in both age and T1D. (See also Figures 4-5).

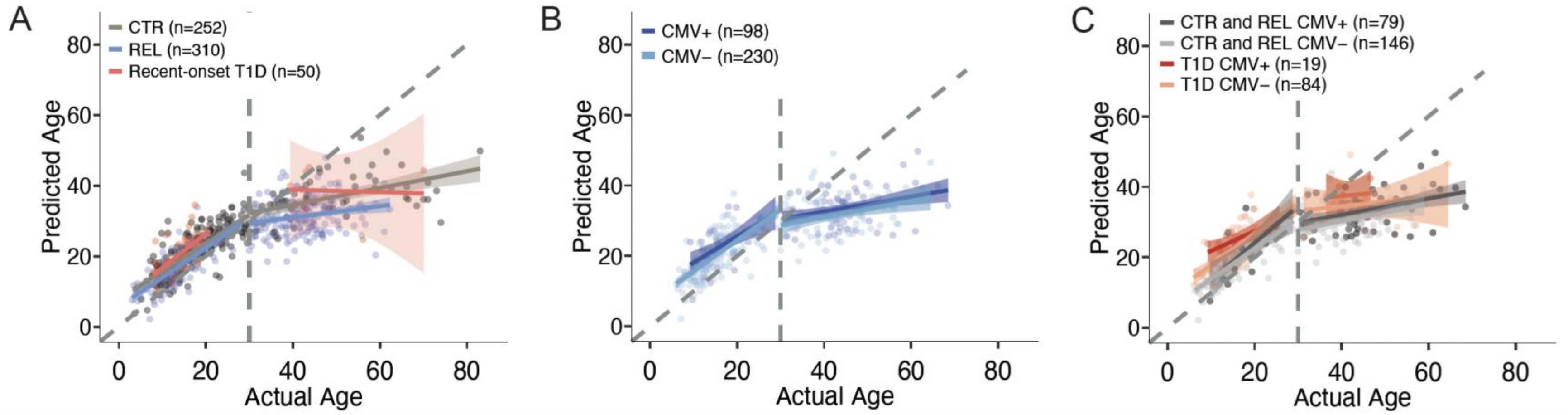

Figure S10. Immune aging model with CMV status. (**A**) Predicted immunological age is shown for all CTR (gray), AAb- REL (blue), and recent-onset T1D (duration < 1 year, red). The relationship between predicted age with chronological age is shown using a piece-wise regression model with a break at chronological age 30. (**B**) The immunological predicted age is shown for all T1D and AAb- individuals combined. Points are colored based on CMV status with CMV positive in dark blue and CMV negative in light blue. The relationship between predicted age with chronological age is shown as in panel A. (**C**) Similar in structure to panel B, but additionally split by cohort. Gray represents AAb- individuals (CTR and REL combined) with CMV+ AAb- in dark gray and CMV- AAb- in light gray, while T1D are in red with CMV+ T1D in dark red vs CMV- T1D in pink. (See also Figure 3).

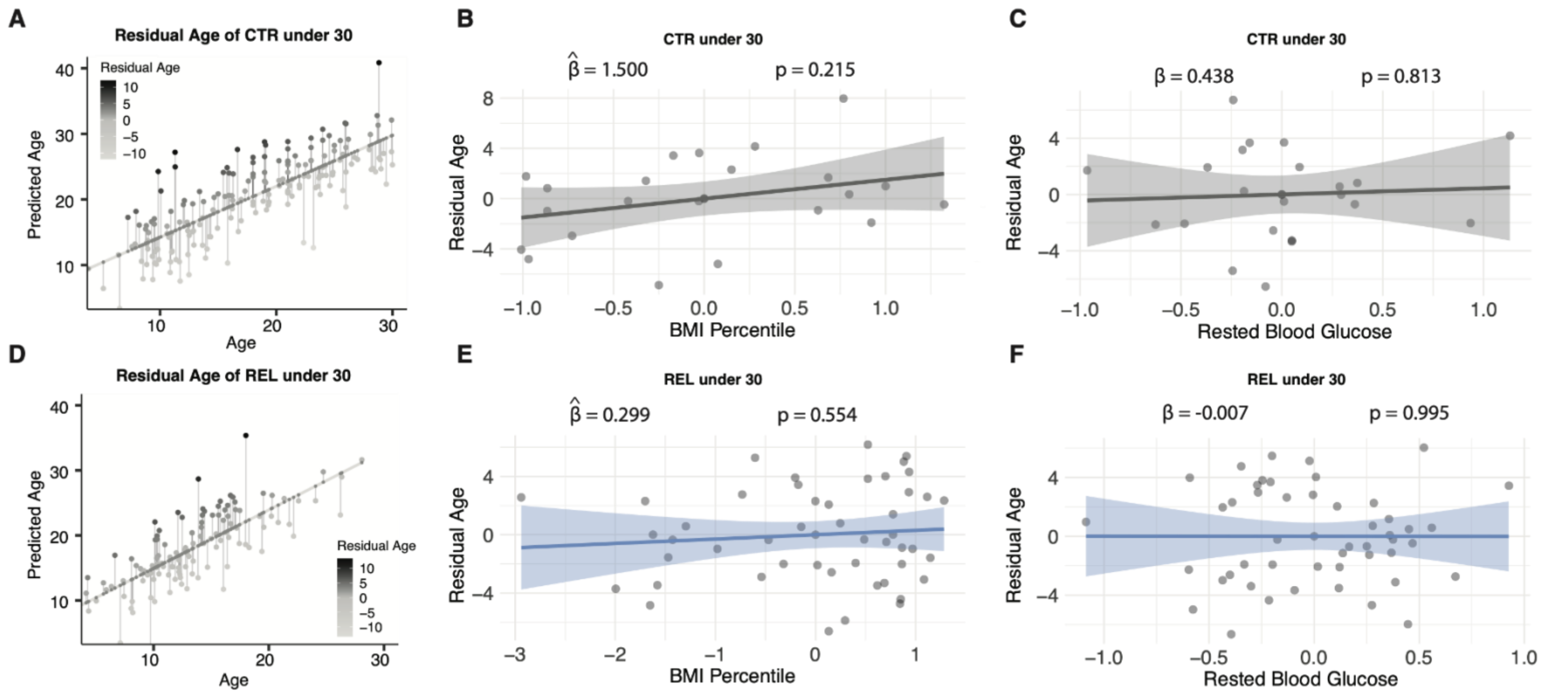

Figure S11. Residual age analysis in AAb- CTR and REL under 30 years of age. (**A**) Residual immunological age is calculated as the residual from a linear regression of predicted age and chronological age for controls. (**B**) The partial regression plot of residual age and BMI percentile is shown along with the standardized regression coefficient and p-value. (**C**) Similar in structure to B for the association between residual age and rested blood glucose. (**D**) Similar in structure to panel A, but for AAb- REL. (**E-F**) similar in structure to panels B and C for AAb- REL. (See also Figure 3).

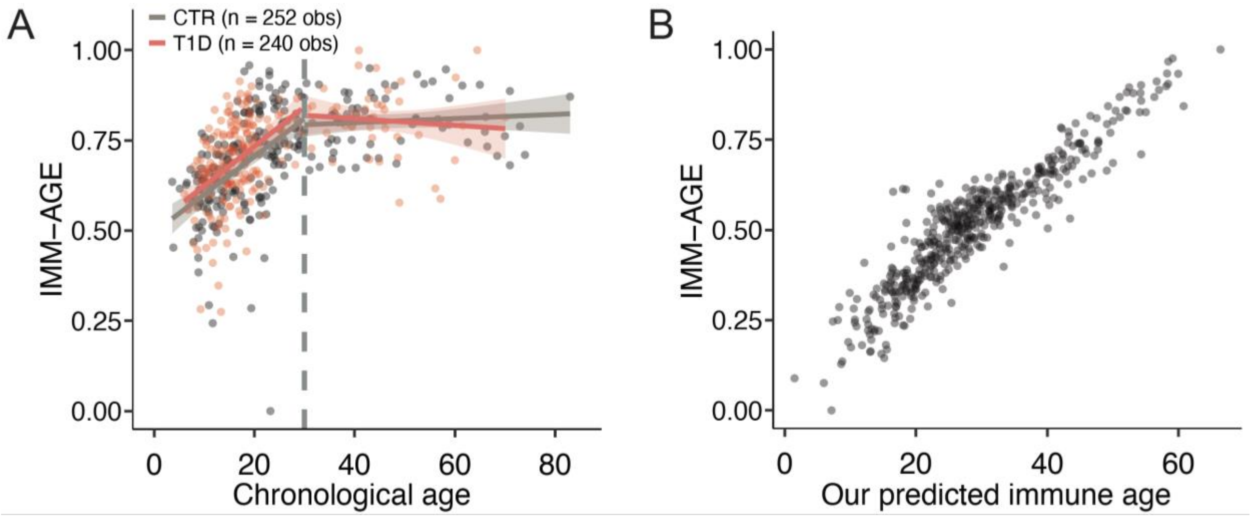

Figure S12. Immune aging model compared to IMM-AGE. (**A**) The IMM-AGE score procedure was applied to all 192 immune phenotypes for T1D and CTR individuals. Points are colored based on cohort. The relationship between IMM-AGE with chronological age is shown using a piece-wise regression model with a break at chronological age 30. T1D individuals under the age of 30 have a significantly larger IMM-AGE score compared to Controls (p=0.022) (**B**) Our predicted immune age is compared to the IMM-AGE score computed using only the 69 features identified by our approach. The Spearman correlation is 0.92.

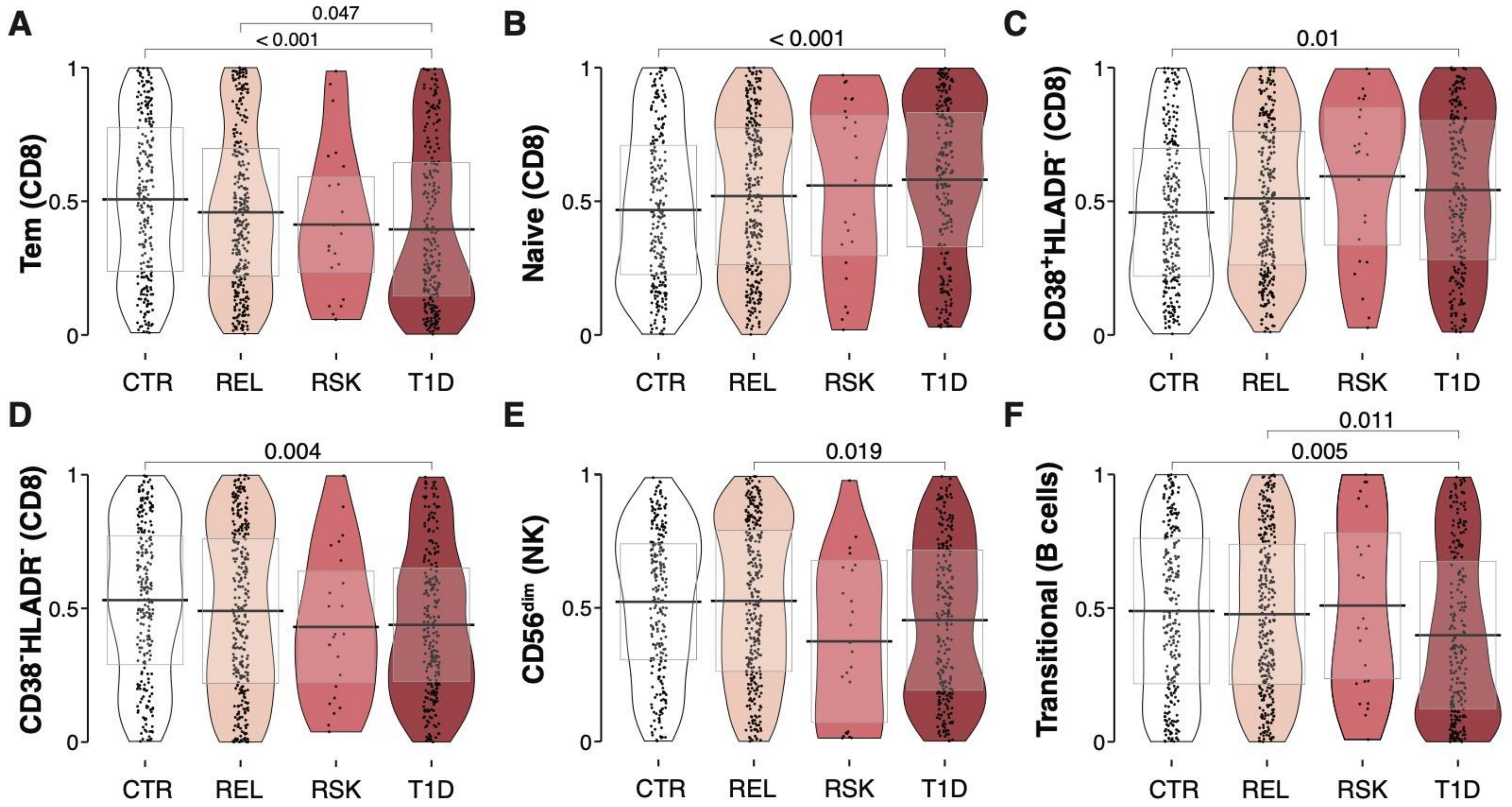

Figure S13. Adaptive immune compartment findings confirming prior literature. Immune subsets from AAb- CTR, AAb- REL, >2 AAb+ RSK, and T1D individuals. Significant p-values shown on graph. Testing was done using a Kruskal Wallis test with a post-hoc Dunn's test and Benjamini-Hochberg multiplicity adjustment. The y-axis represents age-adjusted quantile values.

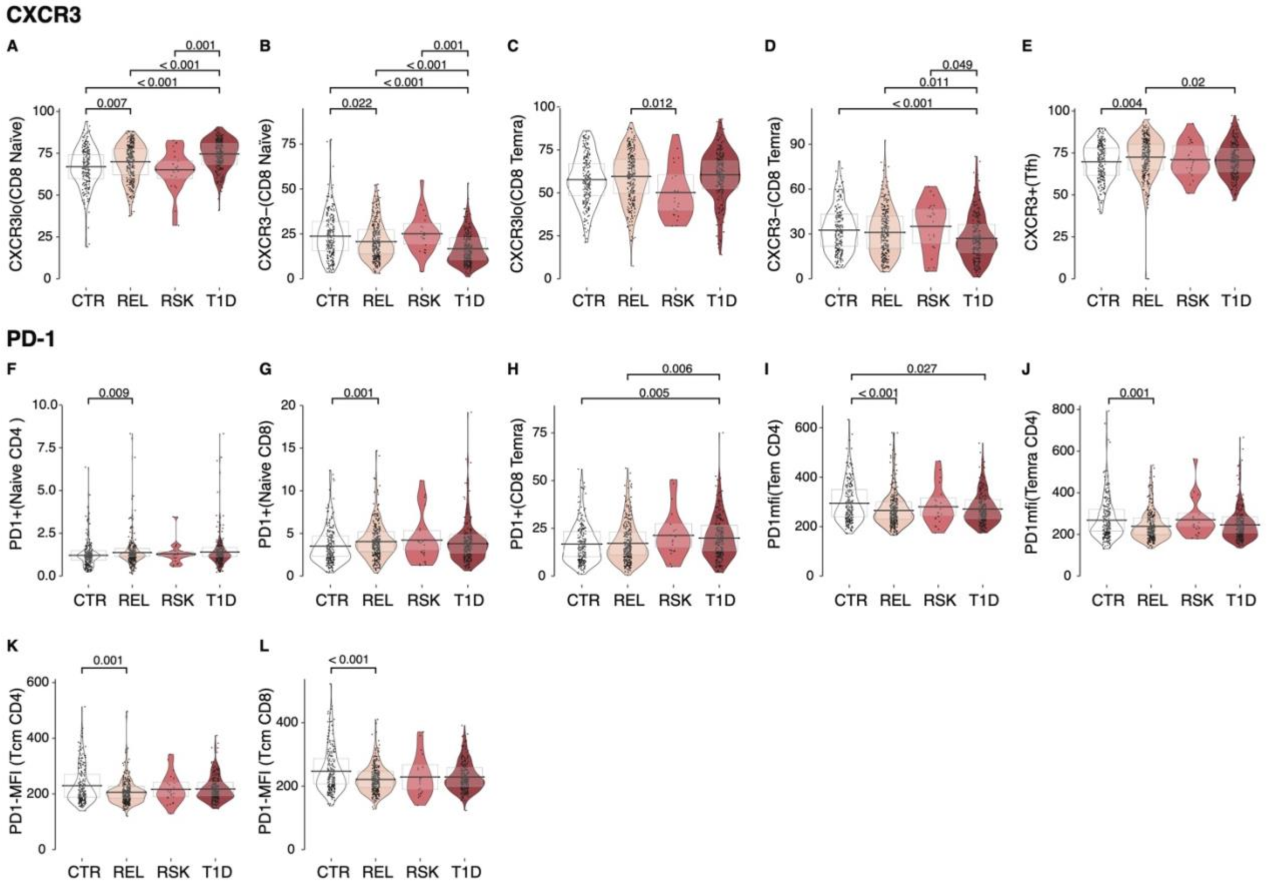

Figure S14. Raw data shows CXCR3 and PD-1 expression is increased on naïve T cells but decreased on memory T cell subsets in AAb- CTR, REL, >2 AAb+ and T1D individuals. (**A-D**) CXCR3 low or negative proportions on CD8 subsets, (**E**) CXCR3 positive proportions of CD4 Tfh cells, (**F-H**) PD-1$^+$ and (**I-L**) PD-1 MFI for each phenotype shown from AAb- CTR, AAb- REL, >2 AAb+ RSK, and T1D individuals. Significant p-values shown on graph. Testing was done using a Kruskal Wallis test with a post-hoc Dunn's test and Benjamini-Hochberg multiplicity adjustment (See also Figure 6).

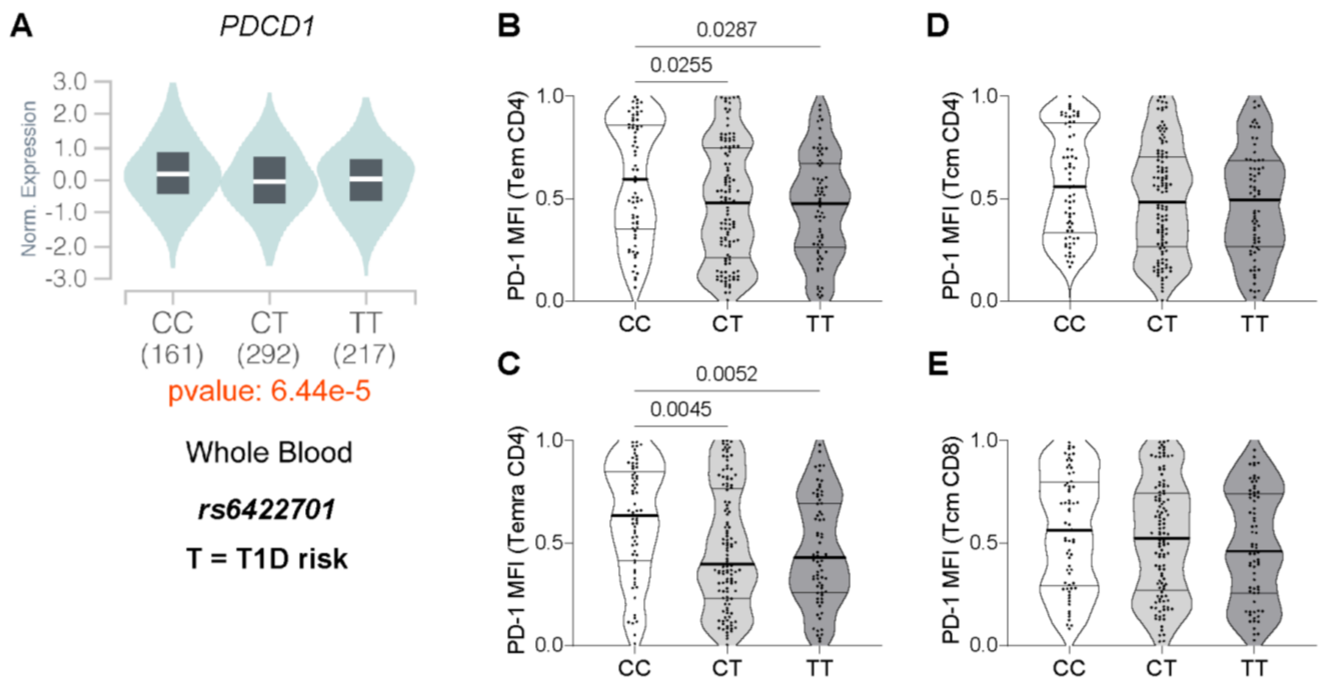

Figure S15. ***PDCD1*** **expression quantitative trait locus (eQTL), rs6422701, associates with PD-1 mRNA and protein expression.** (**A**) Genotype-Tissue Expression project (GTEx) data shows normalized *PDCD1* mRNA expression in whole blood differs according to genotype at rs6422701. In our cohort, the T allele of rs6422701 was overrepresented in the T1D group (See Table S4). Age-adjusted quantile values of PD-1 MFI on (**B**) CD4 Tem, (**C**) CD4 Temra, (**D**) CD4 Tcm, and (**E**) CD8 Tcm of AAb- CTR and AAb- REL according to rs6422701 genotype. Significant p-values shown on graph. Testing was done using a Kruskal Wallis test with a post-hoc Dunn's test.
